# From Primary Cells to Organoids: Expanding the Biological Relevance of In Vitro Cellular Models

**DOI:** 10.3390/cells15191741

**Published:** 2026-09-24

**Authors:** Fengyan Deng, Yan Wang, Luciana A. Castellano, Chongbei Zhao

**Affiliations:** Stowers Institute for Medical Research, Kansas City, MO 64110, USA; fdeng@stowers.org (F.D.); yw2228@stowers.org (Y.W.); lcastellano@stowers.org (L.A.C.)

**Keywords:** primary cells, cell immortalization, induced pluripotent stem cells, iPSC reprogramming, organoids, in vitro models, organ-on-chips, cell characterization

## Abstract

**Highlights:**

**What are the main findings?**

- In vitro models form a continuum from primary cells and established cell lines to iPSC-derived organoids and advanced bioengineered systems, with distinct strengths and limitations in modeling human biology.
- Biological relevance must be balanced with reproducibility, scalability, experimental control, and fit-for-purpose characterization and validation.

**What are the implications of the main findings?**

- Model selection should be driven by the biological question and intended context of use rather than by model complexity alone.
- Standardization, bioengineering, automation, and AI will be important for translating advanced cellular models into robust NAMs for biomedical research and drug development.

**Abstract:**

Advanced in vitro cellular models have become indispensable tools for investigating development, disease mechanisms, drug discovery, regenerative medicine, and precision therapeutics. Recent advances in primary cell isolation, cell immortalization, induced pluripotent stem cell (iPSC) technology, and three-dimensional (3D) organoid culture have substantially improved the physiological relevance and versatility of these experimental systems. However, selecting, developing, and validating appropriate in vitro models remain challenging due to differences in biological complexity, reproducibility, and functionality. In this review, we summarize the major strategies for establishing advanced in vitro cellular models, highlighting the principles underlying primary cell derivation, cell immortalization, iPSC reprogramming, and organoid generation. We further discuss critical factors that influence model development, including cell source selection, signaling pathways, microenvironmental regulation, and tissue self-organization, together with current approaches for characterization and functional validation. Finally, we examine emerging bioengineering technologies, key limitations, and future directions for developing increasingly robust, reproducible, and physiologically relevant in vitro models. This review provides a practical framework for researchers seeking to develop and apply advanced cellular model systems for basic research and translational biomedical applications.

## 1. Introduction

In vitro cellular models are experimental platforms composed of living cells and maintained outside the organism under defined conditions. These models offer to capture specific aspects of cellular, tissue, or disease biology while allowing direct manipulation of the extracellular environment, genetic background, soluble elements, and cellular composition.

The development of cell culture begins with the advancement of microscopy (Figure 1). In the seventeenth century, improvements in lenses allowed the observation of previously invisible structures. Cells, the empty walls of dead plant cells from a cork, was introduced by Robert Hooke in 1665, followed by observation of living microorganisms by Antonie van Leeuwenhoek, the father of microbiology, in 1674. In the nineteenth century, cell theory, a combination of work from Matthias Schleiden, Theodor Schwann, and Rudolf Virchow, set up a foundation for cell culture: all living organisms are composed of cells, cells are the basic structural units of life, and all cells arise from pre-existing cells [1]. The very first cell culture was to culture embryonic frog tissue to study nerve fiber growth by Ross Granville Harrison in 1907 with the hanging drop method [2]. This revolutionary work was considered the beginning of cell culture because it helped establish the idea that cells could remain viable, migrate, and expand processes in vitro. In the next century, cell culture reached several milestones that continuously expanded biological research from simple cell propagation to complex tissue modeling. To name a few, in 1951–1952, HeLa cells from cervical carcinoma tissues were established [3,4], creating the first established human cell line widely used for virology, cancer biology, toxicology, and vaccine development. In 1981, mouse embryonic stem cells (ESCs) from early embryos were derived and used to study development, differentiation and gene editing [5,6]. In 1998, human ESCs, representing the human system, opened new possibilities for regenerative medicine, developmental biology, and disease modeling while also raising human ethical issues [7]. Alternatively, in 2006, Yamanaka showed that differentiated mouse fibroblasts could be reprogrammed into induced pluripotent stem cells (iPSCs) [8], with only four transcription factors, which transformed the field by enabling an individual-specific pluripotent platform. Three years later, Clevers et al. reported the first crypt-villus organoid model generated from single Lgr5-positive intestinal stem cells through self-organization, which led the shift from traditional two-dimensional (2D) culture towards three-dimensional (3D) tissue models [9].

In vitro cellular models include primary cultures, established cell lines, 2D, three-dimensional (3D) organoids, co-culture systems, organ-on-a-chip, and 3D bioprinting platforms. Primary cell culture represents the earliest and most biologically direct form of in vitro cellular modeling. Its historical foundation is often traced to Harrison’s early twentieth-century experiments, in which fragments of embryonic frog tissue were maintained outside the body [2]. This work demonstrated that living animal cells could survive, migrate, and differentiate in an artificial environment, establishing the conceptual and technical basis for modern animal cell culture. Later refinements by researchers such as Carrel and Burrows improved explant culture methods, sterile handling, culture vessels, and nutrient conditions, allowing primary tissues from embryos, organs, tumors, and adult tissues to be maintained more reproducibly in vitro [10]. Primary cells are isolated directly from tissues and therefore retain many features of their original biological context, including tissue-specific morphology, gene expression, differentiation state, signaling behavior, and physiological responses. This makes primary cell culture especially valuable for studying normal cell biology, developmental processes, host–pathogen interactions, toxicology, pharmacology, tissue repair, and disease mechanisms. Primary human cells are particularly important because they provide access to human biology that cannot be fully modeled in animals. However, primary cell culture also showed technical limitations: difficulty in isolation, limited lifespan, heterogeneity, and variability between samples. These challenges promoted the development of immortalized cell lines, which offer greater scalability and reproducibility. The landmark milestone was the HeLa cells, which showed proliferative capacity and the feasibility of long-term human cell propagation [3,4]. The development of established cell lines revealed important biological principles. For example, the finite lifespan of normal human diploid cells [11,12] led to the concept of cellular senescence and immortalized/transformed lines. Since then, many cell lines have become central for research and biomedicine, for example, HEK293 cells for transfection and viral production, and CHO cells for recombinant protein and antibody manufacturing [13].

Building on early primary and established cell culture methods, the development of sterile techniques, defined media, serum, antibiotics, CO_2_ incubators, and standardized culture vessels made 2D culture widely accessible and reproducible. 2D monolayer culture, cells growing on flat surfaces, then became dominant due to its simplicity, scalability, cost-effectiveness, genetic manipulation and compatibility with multiple platforms such as microscopy, drug testing and molecular biology. However, 2D culture also has important limitations: tissue architecture, cell–cell interactions, mechanical cues, polarity, nutrient gradients, and organ-level complexity. As a result, 2D models may poorly predict in vivo physiology, drug responses, and toxicity. These limitations prompted the development of 3D culture to better mimic the in vivo tissue microenvironment.

Organoids are self-organizing 3D cultures from adult stem cells isolated from healthy or patient tissues, or from iPSCs that recapitulate key aspects of native organ architecture and functions. Organoids have now been generated from a wide variety of human organs, including intestine, stomach, liver, pancreas, lung, kidney, brain, retina, prostate, mammary gland, endometrium, and multiple tumor types, enabling tissue-specific studies of development, disease, regeneration, and drug response. Beyond human systems, organoids and organoid-like cultures have also been established across diverse animal species, including rodents, livestock, companion animals, and other emerging model or non-model organisms, expanding their potential applications in comparative biology, veterinary medicine, evolution, conservation, and cross-species disease modeling [14]. Organ-on-chip systems use microfluidic devices to model tissue interfaces, fluid flow, mechanical forces, and organ-level physiology. 3D printing and bioprinting technologies add spatial control by depositing cells, biomaterials, and sacrificial templates into defined architectures, enabling vascular-like networks and engineered tissues with improved perfusion and reproducibility. Together, these approaches extend conventional 2D culture toward more physiologically relevant models for development, disease modeling, toxicity testing, drug discovery, and regenerative medicine.

Common challenges in in vitro cellular models include contamination, genetic instability, and phenotypic drift [15,16]. Microbial contamination from bacteria, fungi, yeast, mycoplasma, viruses, algae, or protists can affect cell growth, metabolism, gene expression and experimental outcomes. Mycoplasma contamination is especially problematic because it can persist undetected and significantly affect cellular behavior. Long-term culture or gene editing methods can also result in genetic instability, including chromosomal abnormalities, copy-number changes, and mutations, especially in immortalized or transformed cell lines. Even primary cells and stem-cell derived models may undergo phenotypic drift, gradually losing tissue-specific functions or differentiation states. Other major limitations include batch-to-batch variability in serum, extracellular matrix products, growth factors, and donor-derived cells. In vitro models may also lack full tissue architecture, vascularization, immune interactions, innervation, mechanical forces, and systemic metabolism, reducing their ability to predict complex in vivo responses. For advanced models such as organoids and organ-on-chip systems, challenges include protocol variability, scalability, cost, assay standardization, quality control, and regulatory validation. Therefore, careful authentication, contamination testing, passage-number control, standardized protocols, appropriate controls, and context-of-use validation are essential for reliable application in basic science, drug discovery, toxicology, and translational medicine.

Together, in vitro cellular models provide controllable and accessible systems for studying cell behaviors, basic biology, translational research, disease modeling, drug discovery, and the development of precision medicine approaches. In this review, we provide a comprehensive review of four major in vitro models—primary cells, immortalized cell lines, iPSCs, and organoids—covering their historical development, biological mechanisms, key establishment strategies, and approaches for characterization and functional validation. This review also provides a practical framework for researchers seeking to develop and apply advanced cellular model systems for basic research and translational biomedical applications (Figure 1).

Rather than viewing the progression from primary cells to cell lines, iPSC-derived systems, and organoids as a linear increase in model quality, this review considers these platforms as complementary models with distinct experimental strengths and limitations. We compare these systems in terms of biological fidelity, variability, maturity, scalability, genetic tractability, functional validation, and intended context of use (Table 1, Graphical Abstract). This fit-for-purpose framework provides a basis for selecting model complexity according to the biological question rather than assuming that increasing complexity inherently improves experimental relevance.

## 2. Primary Cell Derivation and Cell Line Establishment via Immortalization

### 2.1. Overview of the Advantages and Disadvantages of Primary Cells and Immortalized Cells

Primary cells are directly derived from tissues or organs of an organism and cultured under optimized in vitro conditions. A key advantage of primary cells is their ability to closely recapitulate the transcriptomic and proteomic profiles, as well as the physiological functions of their tissue of origin. This high degree of biological relevance makes them a powerful model system for studying cellular physiology and biochemistry, evolutionary processes, virology, disease modeling, drug discovery and screening, vaccine development, and a wide range of other biomedical applications [17,18,19,20]. Despite these advantages, primary cell models are associated with several important limitations. First, because primary cells are directly isolated from tissues, the initial cultures often contain mixed cell populations or distinct cellular subtypes that reflect the heterogeneity of the source tissue. Although optimized culture conditions can be used to enrich specific cell populations, residual heterogeneity frequently persists [21,22]. This cellular heterogeneity can complicate experimental interpretation and reduce consistency between preparations. However, it can also represent an important biological advantage of primary-cell cultures. The retention of multiple cell types or cellular subpopulations may better preserve aspects of the cellular diversity, cell–cell interactions, and microenvironment of the tissue of origin [15,16]. Therefore, whether heterogeneity represents a limitation or an advantage depends on the experimental question: highly enriched populations may be preferable for cell-intrinsic mechanistic studies, whereas heterogeneous cultures may provide greater biological relevance when multicellular interactions are important. This concept is further reflected in more complex in vitro models, such as organoids, which intentionally incorporate cellular diversity and tissue organization to capture additional aspects of in vivo biology. Second, primary cells derived from different donors or animals exhibit inherent biological variability, which can compromise experimental reproducibility. For example, cells from different sources may respond differently to the same stimuli, leading to variable outcomes [15,16]. Third, primary cells possess a finite proliferative capacity and eventually undergo replicative senescence, a phenomenon known as the Hayflick limit [11,12]. However, their proliferative lifespan varies substantially depending on factors such as cell type, species, donor characteristics, developmental stage, isolation procedure, and culture conditions; therefore, a universal passage number cannot be used to define the onset of senescence across primary cell models [23,24,25]. Finally, maintaining the functional phenotype of primary cells in vitro remains challenging, as cellular characteristics and functions can drift over time [26,27]. Accordingly, rather than applying a universal passage-number cutoff, primary cells should ideally be used within a well-characterized and experimentally validated passage range in which the relevant phenotypic and functional properties of the specific cell model are maintained.

Thus, the establishment of immortalized cell lines, which can overcome replicative senescence (the Hayflick limit) while preserving the similar genotype and phenotype of the parental primary cells or tissues, becomes an alternative approach (Table 1) [27]. Primary cells can undergo immortalization either spontaneously or through genetic manipulation. Common strategies include the introduction of telomerase reverse transcriptase (TERT) [28], simian virus 40 (SV40) large T antigen [29,30], or cyclin-dependent kinase 4 (CDK4) [31], which enable cells to bypass senescence and extend their proliferative capacity. Although well-characterized immortalized cell lines can provide greater experimental consistency and scalability than finite primary cultures, immortalization does not necessarily preserve all properties of the parental cells [15,16]. The extent to which genotype, phenotype, differentiation capacity, and physiological functions are retained depends on the cell type and immortalization strategy, and additional changes may arise through clonal selection, genetic or epigenetic adaptation, and prolonged culture [15,16]. Therefore, the biological fidelity and functional stability of immortalized cell lines should be experimentally validated for their intended applications.

### 2.2. Cell Immortalization: Key Considerations and Workflow

Cell immortalization is a powerful approach for overcoming the limited proliferative capacity of primary cells and generating stable cell lines for long-term research applications. Successful immortalization, however, requires more than simply introducing immortalizing factors. The quality of the resulting cell lines is heavily influenced by multiple factors throughout the process, including the selection and characterization of parental primary cells, optimization of culture conditions, choice of immortalization strategy, efficient delivery of immortalization factors, clone selection and expansion, and comprehensive validation of the established cell lines. Careful attention to each of these steps is essential to maximize immortalization efficiency while preserving the molecular, phenotypic, and functional characteristics of the parental cells. This section summarizes the key considerations, common challenges, and best-practice recommendations for establishing reliable and biologically relevant immortalized cell lines.

#### 2.2.1. High-Quality Primary Cells

The selection of high-quality, early-passage parental primary cells is a critical determinant of successful cell immortalization, as it directly influences the genotype, phenotype, and functional stability of the resulting cell lines. Because the biological characteristics of the starting population can strongly influence the properties of the resulting immortalized cells, variability or cellular damage introduced during primary cell derivation can be amplified or further altered during immortalization and subsequent selection.

The derivation of high-quality primary cells depends on several key factors. First, the quality of the starting tissue is fundamental. Fresh tissues are generally preferred, as prolonged ischemia or suboptimal handling can compromise cell viability and induce stress responses [23,32,33]. When fresh samples are not available, well-preserved frozen tissues can serve as an alternative, provided that optimized cryopreservation and thawing protocols are used to preserve cell viability and minimize cellular damage [24,25]. Poor tissue quality may lead to reduced cell yield, altered gene expression profiles, or selective survival of stress-resistant subpopulations [34,35].

Second, efficient and gentle tissue dissociation is essential for the successful isolation of viable single cells or small cell clusters. Enzymatic digestion (e.g., collagenase, trypsin, dispase) and/or mechanical dissociation should be carefully optimized for each tissue type [21,36]. Overly harsh digestion could damage cell membranes, surface proteins, and receptors, whereas insufficient dissociation may reduce cell yield and increase heterogeneity [21]. Importantly, dissociation conditions could influence downstream cell behavior, including proliferation capacity and responsiveness to immortalization [21].

Third, the choice of culture medium plays a central role in supporting cell survival, proliferation, and lineage-specific function. Basal media, serum concentration, growth factors, and supplements (e.g., EGF, FGF, insulin, antioxidants) should be tailored to the specific cell type [23,24,25]. Inappropriate media conditions may induce differentiation, cellular stress, or selection bias, thereby altering cellular composition and reducing the physiological relevance of the model [24].

Prevention of microbial contamination is another critical factor during primary cell derivation. Primary tissues are directly collected from animals or patients and may carry bacteria, fungi, mycoplasma, or other microorganisms originating from the skin, mucosal surfaces, or surrounding environment. Therefore, strict aseptic techniques should be implemented throughout tissue collection, transportation, and processing. Tissues should be collected using sterile instruments, transported promptly in sterile chilled medium, and washed thoroughly with sterile phosphate-buffered saline (PBS) or balanced salt solution supplemented with appropriate antibiotics and antifungal agents to reduce surface contaminants before dissociation [23,24,25]. During the initial establishment of primary cultures, culture media are often supplemented with antibiotics and antifungal agents for the first few passages to minimize microbial contamination while cells recover and expand. Once cultures are established and contamination-free, however, these agents should be gradually removed whenever possible. Prolonged antibiotic use may mask low-level contamination, alter cellular physiology, and promote the emergence of resistant microorganisms. Therefore, long time antibiotics treatment should also be avoided.

In addition, physical and environmental parameters, including extracellular matrix (ECM) coatings (e.g., collagen, laminin, Matrigel), oxygen tension, temperature, CO_2_ levels, seeding density, and passaging methods, significantly influence cell behavior. For example, reduced oxygen levels (physiological hypoxia) may better preserve stemness or tissue-specific functions in certain cell types [37,38]. Appropriate seeding density is critical to maintain optimal cell–cell interactions and prevent stress associated with under- or over-confluence, while gentle and well-controlled passaging methods help preserve cell viability and functional integrity [39,40]. Conversely, overgrowth, excessive cellular stress, or repeated harsh dissociation can impair proliferation, alter cellular phenotypes, and accelerate senescence [39,40]. Overall, suboptimal culture conditions can lead to phenotypic drift, reduced proliferative capacity, or premature senescence [39,40].

Finally, comprehensive characterization of primary cells is essential prior to initiating immortalization. Cell identity should be confirmed using immunofluorescence staining or other marker-based approaches, while functional assays (e.g., differentiation capacity) can validate cell-specific properties [24]. Genetic stability should be assessed through chromosome counting or karyotyping, and routine mycoplasma testing is necessary to ensure culture health [24]. Once primary cells are thoroughly validated, immortalization should be initiated as early as possible to minimize the accumulation of cellular stress, senescence-associated changes, and genetic alterations. Cells at later passages often exhibit reduced proliferation, altered morphology, and compromised functionality, which can negatively impact immortalization efficiency and the quality of derived cell lines [24].

#### 2.2.2. Choice of Immortalization Strategy

The selection of an appropriate immortalization strategy is a critical step in establishing stable and biologically relevant cell lines. Different immortalization methods vary substantially in their efficiency, impact on cellular physiology, genetic stability, and ability to preserve the characteristics of the parental primary cells [22,41]. Therefore, the choice of strategy should be guided by the cell type, species, intended downstream applications, and the level of physiological fidelity required.

Currently, cell immortalization strategies can be broadly categorized into spontaneous immortalization and induced immortalization [23,24,25] (Figure 2). Spontaneous immortalization occurs naturally during long-term culture through the accumulation of genetic and epigenetic alterations that enable cells to bypass senescence [23]. Although spontaneous immortalization has been reported in certain rodent and tumor-derived cells [22], it is relatively rare in human primary cells and is often associated with unpredictable genetic changes and phenotypic alterations [42].

In contrast, induced immortalization involves deliberate manipulation of pathways associated with telomere maintenance, cell-cycle regulation, or senescence. Among these approaches, telomerase reverse transcriptase (TERT)-mediated immortalization is one of the most widely used strategies. TERT maintains telomere length and prolongs proliferative lifespan while generally preserving cellular phenotype and function more effectively than oncogene-based methods [22,43,44]. However, TERT expression alone may not be sufficient for immortalization in certain cell types, particularly those with strong senescence checkpoints [22,24,43,44].

Viral oncogene-mediated immortalization, including the use of simian virus 40 (SV40) large T antigen or human papillomavirus (HPV) E6/E7 proteins, is highly efficient and widely applied [22,25,41,45,46]. These oncogenes promote continuous proliferation primarily through inhibition of tumor suppressor pathways such as p53 and retinoblastoma protein (pRb). While effective, these approaches may substantially alter cell-cycle regulation, genomic stability, and cellular physiology, potentially reducing the physiological relevance of the resulting cell lines [22,41].

Alternative approaches targeting cell-cycle regulators, such as cyclin-dependent kinase 4 (CDK4), Cyclin D1, or BMI1, have also been used to bypass senescence while minimizing transformation-associated effects. In many cases, combinatorial strategies, such as TERT combined with CDK4 or HPV E6/E7, provide higher immortalization efficiency and improved long-term proliferation across diverse cell types [22,24,41,47].

Importantly, no single immortalization strategy is universally optimal. Highly proliferative methods may increase immortalization efficiency but also elevate the risk of genomic instability, phenotypic drift, or transformation-like properties. Therefore, careful optimization and extensive downstream characterization are essential to ensure that immortalized cell lines retain the desired biological characteristics of the parental primary cells.

#### 2.2.3. Delivery Methods for Immortalization Factors

Efficient delivery of immortalization factors into primary cells is a critical determinant of successful cell line establishment. Because primary cells are often sensitive, slow proliferating, and difficult to transfect, the choice of delivery method can substantially influence transgene expression efficiency, cell viability, genomic stability, and ultimately the quality of the resulting immortalized cell lines [48,49]. Therefore, delivery strategies should be carefully selected and optimized according to the cell type, species, proliferative status, and immortalization factors being introduced (Figure 2).

Viral vector-mediated gene delivery remains one of the most commonly used approaches for cell immortalization due to its high transduction efficiency and stable transgene expression [22]. Among these methods, lentiviral vectors are widely applied because they can efficiently infect both dividing and non-dividing cells and support stable genomic integration of immortalization factors such as TERT, SV40 large T antigen, HPV E6/E7, or CDK4 [24,25,50]. Retroviral vectors are also frequently used; however, they primarily infect actively dividing cells and are therefore less effective in quiescent or slowly proliferating primary cells [51]. Viral delivery methods generally provide robust and long-term expression, but they may introduce insertional mutagenesis, variable copy numbers, or unwanted alterations in cellular physiology [52,53,54].

Non-viral delivery approaches offer alternative strategies with reduced biosafety concerns and lower risks of random genomic integration [48,55]. Lipid-based transfection methods, such as Lipofectamine or FuGENE reagents, are simple and convenient but often exhibit low efficiency in primary cells, particularly in stem cells or difficult-to-transfect cell types [49]. Electroporation and nucleofection, which transiently permeabilize the cell membrane using electrical pulses, can significantly improve delivery efficiency across a wide range of primary cells and species [56]. These approaches are especially valuable for hard-to-transfect cells, although excessive electrical stress may reduce cell viability and require careful optimization of electroporation parameters.

Transposon-based systems, including the piggyBac and Sleeping Beauty systems, have emerged as powerful tools for stable immortalization factor delivery [57]. These systems enable relatively efficient genomic integration with larger cargo capacity and lower cost compared with viral vectors [57]. In particular, the piggyBac system has been widely used for stable gene expression in pluripotent stem cells and primary cells due to its high transposition efficiency and ability to excise transgenes without leaving genomic footprints under certain conditions [57].

Importantly, delivery procedures themselves can induce cellular stress, DNA damage, or transient phenotypic alterations. Therefore, optimization of transduction or transfection conditions, including vector dosage, exposure time, cell density, and recovery process, is crucial to maximize immortalization efficiency while minimizing adverse effects on cell health and biological function [22].

#### 2.2.4. Post-Immortalization Selection, Expansion, and Clone Selection

Following the delivery of immortalization factors, careful post-immortalization selection and expansion are essential to establish stable, biologically relevant, and reproducible immortalized cell lines. At this stage, cells that have successfully incorporated and expressed immortalization factors must be enriched while minimizing the selection of abnormal or excessively transformed populations. The strategies used during this phase can substantially influence the genotype, phenotype, and long-term stability of the resulting cell lines.

Selection of immortalized cells is commonly achieved using antibiotic resistance markers, fluorescence-based reporters, metabolic selection systems, or combinations of these approaches [58]. Antibiotic selection, such as puromycin, neomycin, hygromycin, or blasticidin resistance, is widely used to enrich transgene-positive cells [58]. Before selection, a kill curve should be performed in the parental primary cells to determine the minimum antibiotic concentration required to eliminate non-transduced cells within an appropriate timeframe while minimizing unnecessary cytotoxicity to successfully transduced cells. Selection conditions should then be carefully optimized, as excessive selection pressure can induce cellular stress and preferentially enrich highly resistant but biologically abnormal subpopulations [58]. Similarly, fluorescence-based enrichment using fluorescent reporters and flow cytometry enables rapid isolation of transgene-positive cells, although harsh sorting conditions may negatively affect sensitive primary cells [58]. Following initial selection, immortalized cells are typically expanded either as heterogeneous bulk populations or as clonally derived cell lines [22,43,58]. Bulk populations may better preserve the natural heterogeneity of the parental cells and reduce clonal selection bias. However, heterogeneous populations can also exhibit variable growth rates, transgene expression levels, and functional properties over time. In contrast, single-cell clone isolation enables the identification of stable clones with desired characteristics, including consistent morphology, robust proliferation, stable transgene expression, and preserved cellular functions. Common clone isolation approaches include limiting dilution, fluorescence-activated cell sorting (FACS), cloning cylinders, and manual colony picking.

Clone selection should not rely solely on rapid proliferation [43,58,59]. Excessively fast-growing clones may harbor substantial genomic abnormalities, altered signaling pathways, or transformation-associated phenotypes. Instead, multiple parameters should be evaluated during clone screening, including cell morphology, proliferation kinetics, genomic stability, expression of lineage-specific markers, differentiation potential, and maintenance of key physiological functions [41,58]. Importantly, comparisons with early-passage parental primary cells are essential to determine how closely immortalized clones retain the biological characteristics of the original cells [24].

Because both donor-specific characteristics and clonal selection can influence the properties of established cell lines, biological replication should also be considered during cell-line development and validation [15,16]. When feasible and relevant to the intended application, independently derived primary-cell preparations from multiple donors or animals can help determine whether observed characteristics are reproducible across biological backgrounds rather than specific to an individual donor [15,16]. Similarly, when clonally derived immortalized lines are used, characterization of multiple independently derived clones can help distinguish reproducible properties associated with the immortalization strategy from clone-specific genetic, epigenetic, or phenotypic alterations [15,16]. The extent of donor and clonal replication should be determined according to the intended application and experimental question.

During expansion, culture conditions should be carefully controlled to minimize cellular stress and phenotypic drift [60]. Over-confluence, repeated harsh dissociation, prolonged culture without passaging, and excessive passage numbers may promote genetic instability or functional alterations [60,61]. Early banking of validated immortalized populations and selected clones is therefore highly recommended to preserve low-passage stocks and reduce long-term culture-associated changes. In addition, routine cryopreservation at appropriate intervals throughout the immortalization process is strongly encouraged [59]. This practice provides critical backup stocks in case of contamination, phenotypic drift, genetic instability, or unexpected culture loss, thereby minimizing the need to repeat the lengthy and labor-intensive immortalization procedure.

In addition, long-term monitoring of immortalized cells is critical. Even after successful establishment, immortalized cell lines may gradually accumulate chromosomal abnormalities, epigenetic alterations, or functional drift during continuous culture [60,61]. Routine quality control measures, including morphology assessment, growth analysis, mycoplasma testing, karyotyping, and marker validation, should therefore be performed regularly to ensure the reliability and reproducibility of the established cell line [58,60,61].

#### 2.2.5. Characterization and Validation

Comprehensive characterization and validation are essential following immortalized cell line establishment to ensure that the resulting cells retain the identity, stability, and functional properties of the parental primary cells [24,59]. Because immortalization and long-term culture may introduce genetic, phenotypic, or functional alterations, rigorous validation should be routinely performed before experimental application and throughout continuous culture [24,58,59].

#### 2.2.6. Cell Identity Verification and Authentication

Verification of cell identity is a fundamental step in immortalized cell line characterization. Initial assessment commonly includes evaluation of cellular morphology, growth pattern, and adherence characteristics using phase-contrast microscopy [15,24,43]. Comparison with the parental primary cells can help determine whether the immortalized cells retain expected morphological features [24,58]. In addition, cell-type-specific marker expression should be carefully examined using multiple complementary approaches, including immunostaining, flow cytometry, quantitative PCR (qPCR), Western blotting, and transcriptomic profiling such as RNA sequencing (RNA-seq) [41,59,62]. These analyses are critical to confirm lineage identity and determine whether immortalization alters the molecular characteristics of the original cells. Importantly, marker panels should include both positive lineage markers and negative markers to exclude contamination or unintended lineage conversion [62].

#### 2.2.7. Genetic and Genomic Stability

Assessment of genetic and genomic stability is particularly important because immortalization and extended in vitro culture may promote chromosomal abnormalities or genetic drift [43]. Conventional chromosome counting and karyotyping remain widely used methods to evaluate large-scale chromosomal integrity and aneuploidy [15,24,25,59]. More advanced genomic analyses, including single nucleotide polymorphism (SNP) arrays, comparative genomic hybridization (CGH), or whole-genome sequencing, may be necessary for sensitive applications or long-term cultured cell lines [63]. These approaches can identify copy number variations, structural rearrangements, mutations, or insertional effects associated with immortalization factor integration.

#### 2.2.8. Confirmation of Immortalization and Functional Validation

Successful immortalization should be experimentally verified rather than inferred solely from prolonged culture. Long-term proliferation analysis, including extended growth curves and population doubling assays, is commonly used to demonstrate bypass of replicative senescence and sustained proliferative capacity [24,25]. Additional confirmation can include senescence-associated β-galactosidase staining to evaluate senescence status and assessment of immortalization factor expression, such as TERT, SV40 large T antigen, HPV E6/E7, or CDK4 expression. Depending on the immortalization strategy used, telomerase activity assays or telomere length analysis may also provide valuable supporting evidence [43,64,65]. Importantly, growth characteristics of immortalized cells should be compared with those of parental primary cells, including proliferation rate, morphology, population doubling time, and long-term culture stability [15,24,43]. Excessively rapid growth or abnormal morphology may indicate transformation-associated alterations rather than successful physiological immortalization [43].

Beyond molecular characterization, functional validation is essential to determine whether immortalized cells retain the biological properties of the parental primary cells [22,24,66]. Functional assays should be selected according to the specific cell type and intended downstream applications. Examples include differentiation assays for stem or progenitor cells [66], contractility assays for muscle cells [24], secretion assays for endocrine cells [67], barrier integrity assays for epithelial cells [68], and electrophysiological analyses for neuronal cells [69].

#### 2.2.9. Contamination Testing

Routine contamination testing is essential throughout immortalized cell line establishment and maintenance. Mycoplasma contamination is especially problematic because it can alter cellular metabolism, proliferation, gene expression, and experimental outcomes without obvious morphological changes [70]. Therefore, regular mycoplasma screening should be incorporated into standard quality control procedures. In addition, bacterial and fungal contamination should be routinely monitored during culture [15,16].

### 2.3. Conclusions

Collectively, successful establishment of biologically relevant immortalized cell lines requires careful experimental design, rigorous optimization, and comprehensive validation throughout the entire workflow (Figure 2).

## 3. iPSC Reprogramming

### 3.1. Introduction: Historical Development of iPSC Technology

#### 3.1.1. From Blastocyst to Stem Cell Line: The Origin of ESCs

Embryonic stem cells (ESCs) originate from the inner cell mass (ICM) of the blastocyst, which develops into embryonic tissues after fertilization, while the trophoblast forms extra-embryonic structures like the placenta. In 1981, the first mouse ESC (mESC) lines were derived from the ICM [5], demonstrating that pluripotent cells can be maintained in vitro under appropriate culture conditions. mESCs are capable of differentiating into derivatives of all three embryonic germ layers. They maintain a normal diploid karyotype and can contribute to all adult tissues, including the germline, following injection into the blastocyst.

Human ESCs (hESCs) were first successfully established in 1998 following many years of experimental challenges [7]. In this landmark study, Thomson et al. reported the derivation of the first hESC lines from donated fresh and frozen human embryos. In their study, 14 inner cell masses (ICMs) were isolated from 20 embryos, leading to the successful establishment of five independent hESC lines (H1, H7, H9, H13, and H14) [7].

Although ESCs have significant potential for studying development, disease modeling, and regenerative medicine, their therapeutic application is limited by ethical, immunological, and technical challenges. The derivation of hESCs involves the destruction of human embryos, leading to major ethical concerns [71,72]. In addition, hESCs are not inherently patient-specific, which can limit their utility for modeling diseases influenced by individual genetic backgrounds. These ethical and biological limitations, together with the need for patient-specific pluripotent models, motivated the development of alternative approaches, most notably the generation of induced pluripotent stem cells (iPSCs) from somatic cells. Patient-specific iPSCs can be differentiated into relevant cell types, providing genetically matched in vitro models for investigating disease mechanisms and cellular phenotypes [73].

#### 3.1.2. From Somatic Cells to Pluripotency: The Origin of hiPSCs

Early studies demonstrated that somatic cells could acquire pluripotency through fusion with embryonic stem cells [74,75] or by somatic cell nuclear transfer (SCNT) [76], suggesting that oocytes and ESCs contain factors capable of inducing pluripotent states in differentiated cells. This concept led to attempts to induce pluripotency in somatic cells through exposure to factors involved in the maintenance of pluripotency in both early embryos and ESCs.

A major breakthrough occurred in 2006 when Shinya Yamanaka and Kazutoshi Takahashi showed that ectopic expression of four transcription factors, namely, OCT4, SOX2, KLF4, and c-MYC, known as “Yamanaka factors”, could reprogram mouse fibroblasts into induced pluripotent stem cells (iPSCs) [8]. These cells closely resembled ESCs in morphology, gene expression, epigenetic state, and differentiation potential.

In 2007, multiple groups independently reported the successful generation of human iPSCs (hiPSCs) from fibroblasts using similar combinations of pluripotency-associated transcription factors [77,78,79]. hiPSCs displayed characteristics comparable to hESCs, including expression of pluripotency markers and the ability to differentiate into derivatives of all three germ layers. The development of patient-specific iPSCs rapidly expanded applications in disease modeling, drug discovery, and regenerative medicine [80].

Since then, iPSC technology has been successfully applied across numerous species [81,82,83,84], demonstrating that the core transcriptional network regulating pluripotency is broadly conserved across species, although some organisms exhibit challenges to reprogramming and require additional modifications to induce pluripotency [85,86].

### 3.2. Mechanisms Underlying Somatic Cell Reprogramming

Cell differentiation and somatic cell reprogramming represent two opposing yet closely related biological processes. During embryonic development, pluripotent stem cells progressively differentiate into specialized somatic cell types through coordinated transcriptional and epigenetic regulation. In contrast, somatic cell reprogramming reverses this process by erasing lineage-specific programs and re-establishing pluripotency. During differentiation, pluripotent stem cells acquire somatic cell identity through the establishment of epigenetic memory and extensive reorganization of chromatin architecture, resulting in the repression of pluripotency-associated genes and activation of lineage-specific gene expression [87]. Reprogramming somatic cells into induced pluripotent stem cells (iPSCs) requires the erasure of these somatic epigenetic features and the re-establishment of pluripotency through extensive remodeling of gene expression, chromatin structure, DNA methylation, metabolism, and signaling pathways [73]. Consequently, induction of pluripotency has been proposed to partially recapitulate developmental processes in reverse [73,88,89,90]. Reprogramming proceeds through two distinct phases: an early phase marked by silencing of somatic genes and activation of early pluripotency-associated genes, followed by a late phase in which the core pluripotency regulatory network is established. In fibroblasts, mesenchymal-to-epithelial transition (MET) represents a critical step in this process [91].

The transcription factors OCT4, SOX2, KLF4, and c-MYC (OSKM) are the principal regulators of reprogramming [8]. These factors act synergistically to remodel the somatic cell state. They suppress fibroblast-specific gene expression while activating pluripotency-associated transcriptional programs, resulting in global transcriptomic reconfiguration [92,93,94,95,96]. OCT4 contributes to the establishment and maintenance of pluripotency-associated chromatin accessibility; SOX2 acts as a potent pioneer factor capable of binding closed chromatin and facilitating epigenetic remodeling; KLF4 promotes enhancer–promoter interactions; and c-MYC enhances cell-cycle progression and proliferation [96,97,98,99]. Additionally, NANOG and LIN28 have been shown to synergize with OSKM to enhance reprogramming efficiency [96]. Together, these factors cooperate to drive pluripotency acquisition.

Importantly, OSKM cooperate closely to orchestrate global transcriptional reprogramming, exemplified by their coordinated induction of MET, a critical early event in fibroblast reprogramming and the acquisition of an epithelial-like state during the reprogramming of diverse somatic cell types into iPSCs [91]. During MET, cells transition from a motile mesenchymal state to an epithelial phenotype characterized by increased expression of E-cadherin and EPCAM and repression of mesenchymal regulators [100,101]. This process is directly regulated by reprogramming factors and is essential for establishing the epithelial state that supports stable pluripotency.

Successful reprogramming also requires extensive chromatin remodeling and epigenetic reconfiguration. Chromatin accessibility changes dynamically, with somatic loci becoming repressed and pluripotency-associated regions progressively opening [102,103,104]. Histone modifiers such as EZH2 promote reprogramming, whereas DOT1L acts as a barrier, and pharmacological modulation of these pathways can enhance reprogramming efficiency [105,106,107]. In parallel, DNA methylation patterns established during differentiation are reversed through TET-mediated DNA demethylation, leading to activation of pluripotency genes and facilitating MET [108,109,110].

Despite these molecular changes, reprogramming remains inefficient and heterogeneous, with only a small fraction of cells successfully reaching pluripotency [111,112]. Moreover, iPSCs often retain residual somatic-cell epigenetic signatures and may acquire genetic and mitochondrial mutations during reprogramming, contributing to variability in differentiation potential and cellular function [113,114,115,116].

Overall, pluripotency induction emerges from coordinated transcriptional, epigenetic, and structural remodeling driven by the core reprogramming factors OCT4, SOX2, KLF4, and c-MYC, with NANOG and LIN28 further enhancing reprogramming efficiency, enabling the stable conversion of somatic cells into a pluripotent state.

### 3.3. Cellular Reprogramming Strategies

#### 3.3.1. Reprogramming Factors

The choice of reprogramming factors is a critical determinant of iPSC generation. The classical OSKM cocktail (OCT4, SOX2, KLF4, and c-MYC) remains one of the most widely used approaches [117]. However, alternative combinations have been explored to improve reprogramming efficiency and address specific barriers associated with the starting cell population. For example, OSNL (OCT4, SOX2, NANOG, and LIN28) was shown to be sufficient for reprogramming human somatic cells [79,118] while subsequent studies demonstrated that adding KLF4 and c-MYC to this combination could further enhance reprogramming efficiency [119]. Notably, the six-factor OSKMNL cocktail has been shown to enhance reprogramming efficiency and enable the generation of iPSCs from senescent and centenarian donor cells, which represent challenging starting populations for iPSC generation [119,120]. The evolution of these factor combinations reflects advances in understanding the molecular barriers to reprogramming and efforts to improve the efficiency and applicability of iPSC generation.

The optimal combination of factors also depends on the starting cell type, as endogenous expression of pluripotency-associated genes can reduce the need for exogenous factors. For example, melanocytes can be reprogrammed without SOX2 [121], umbilical cord blood stem cells require only OCT4 and SOX2 [122,123], and neural stem cells can be reprogrammed with OCT4 alone [124]. These findings demonstrate the flexibility of reprogramming strategies and highlight the importance of tailoring factor combinations to specific cell types and applications.

#### 3.3.2. Reprogramming Factor Delivery Methods

Multiple strategies have been developed to deliver reprogramming factors and induce pluripotency, each differing in efficiency, safety, and suitability for clinical applications. Viral vector-based approaches were among the first successful methods for iPSC generation (Figure 3). Lentiviral vectors efficiently deliver OSKM factors and achieve robust reprogramming by integrating into the host genome, enabling sustained transgene expression [125,126]. However, genomic integration poses significant risks, including insertional mutagenesis, transgene reactivation, and tumorigenicity, particularly due to the oncogenic properties of c-Myc [127].

To overcome these limitations, non-integrating viral systems have been developed (Figure 3) [128]. These vectors are progressively cleared during iPSC proliferation, enabling reprogramming without permanent OSKM integration or genomic disruption. For example, Sendai virus, a non-integrating RNA virus that replicates exclusively in the cytoplasm, has emerged as one of the most widely used and safest viral reprogramming methods. Sendai virus enables efficient reprogramming without the risks of insertional mutagenesis, residual transgene expression, or genomic disruption, making it particularly attractive for clinical-grade iPSC production [73,129,130].

Non-viral approaches have gained considerable attention because they avoid the risks associated with viral vectors (Figure 3). Episomal plasmids allow transient expression of reprogramming factors without genomic integration and have become commonly used for clinical-grade iPSC generation [131]. Other DNA-based approaches include the PiggyBac transposon system. Although PiggyBac transposons can be excised following reprogramming, concerns remain regarding incomplete excision, insertional mutations, and transgene reactivation [132,133], DNA-based nonviral methods generally exhibit lower reprogramming efficiencies than viral systems.

RNA-based reprogramming represents another promising strategy (Figure 3). Delivery of modified mRNAs encoding reprogramming factors can generate iPSCs without genomic integration while achieving relatively high efficiency and rapid kinetics [134]. However, repeated transfections are required due to the short half-life of mRNA, and exogenous RNA can trigger innate immune responses, needing additional modifications to improve stability and reduce immunogenicity. An alternative RNA-based approach uses self-replicating RNA (srRNA) replicons, such as ReproRNA™-OKSGM (STEMCELL), which encode multiple reprogramming factors (OCT4, KLF4, SOX2, GLIS1, and c-MYC) within a single vector. Unlike conventional mRNA, srRNA persists long enough to enable efficient reprogramming following a single transfection while remaining non-integrating. This approach supports feeder-free generation of iPSCs with high reprogramming efficiency, robust pluripotency marker expression, normal karyotype, and the capacity to differentiate into derivatives of all three germ layers [135].

Protein-based reprogramming further minimizes genetic risks by directly delivering recombinant reprogramming factors into cells, thereby avoiding the introduction of exogenous nucleic acids altogether (Figure 3) [136,137]. Although this approach offers excellent safety, its low efficiency and technical complexity currently limit widespread application.

MicroRNA (miRNA)-mediated reprogramming has also emerged as an alternative strategy (Figure 3). Various combinations of miRNAs can be used to activate the endogenous pluripotency gene networks [138,139]. Because individual miRNAs can target hundreds of transcripts, miRNA-based approaches can induce broad transcriptional changes without introducing exogenous transcription factors.

Alternatively, pluripotency can be achieved using combinations of small-molecule compounds that regulate key signaling pathways and epigenetic modifiers [140]. This chemical reprogramming approach is particularly appealing because of its simplicity and scalability [140,141]. Moreover, integrating small molecules with transcription factor-based methods may increase reprogramming efficiency and accelerate the generation of iPSCs compared with transcription factor-based approaches alone [142,143].

Overall, substantial progress has been made in developing safer and more efficient reprogramming technologies. While integrating viral vectors provide high reprogramming efficiency, concerns regarding genomic modification have shifted attention toward non-integrating approaches such as Sendai virus, episomal plasmids, and mRNA-based methods, which currently represent the most promising platforms for clinical applications [144,145]. Continued optimization of RNA-, protein-, miRNA-, and small-molecule-based strategies will be essential for improving the safety, efficiency, and translational potential of iPSC technology.

The optimal reprogramming strategy should be selected according to the intended application rather than reprogramming efficiency alone. For clinical applications, integration-free delivery systems, such as Sendai virus, episomal plasmids, and modified mRNA, are generally preferred because they minimize the risk of insertional mutagenesis and residual transgene expression. In contrast, for basic research or the establishment of iPSC systems in non-model species, integrating viral vectors may remain valuable because of their robust transgene expression and high reprogramming efficiency. Therefore, selecting an appropriate delivery platform requires balancing efficiency, genomic safety, technical feasibility, and the biological characteristics of the starting cell type. This balancing is typically guided by distinct prioritization criteria: clinical applications strictly prioritize genomic safety over efficiency, whereas basic research or pilot studies in difficult-to-reprogram cells may prioritize high efficiency and technical feasibility to overcome initial biological barriers.

### 3.4. Determinants of Reprogramming Efficiency

#### 3.4.1. Cell Resources

The choice of starting cell type is a major determinant of iPSC reprogramming efficiency, kinetics, and the quality of the resulting pluripotent cell lines (Table 2) [146,147]. Different somatic cell types exhibit distinct proliferative capacities, epigenetic landscapes, endogenous expression of pluripotency-associated genes, metabolic states, and susceptibility to reprogramming, all of which influence the efficiency and reproducibility of iPSC generation. Highly proliferative cells, such as keratinocytes [148], cord blood cells [147], and certain progenitor populations [147,149], generally reprogram more efficiently and rapidly than terminally differentiated cells, partly because of their active cell cycle, permissive chromatin configuration, and endogenous expression of factors associated with stemness [147,149]. In contrast, mature fibroblasts [148] and other highly differentiated cells [147] often require longer reprogramming periods and exhibit lower conversion efficiencies due to more stable lineage-specific epigenetic programs [78,146].

Beyond intrinsic cellular characteristics, donor-related variables also substantially influence reprogramming outcomes [114,150]. Donor age, tissue source, disease status, genetic background, and previous environmental exposures can all affect cellular senescence, DNA damage accumulation, mitochondrial function, and epigenetic memory, thereby influencing both reprogramming efficiency and the differentiation potential of the resulting iPSC lines [114,146,150]. Therefore, careful selection of healthy, low-passage starting cells with high viability and minimal cellular stress is essential for successful reprogramming. Importantly, the optimal cell source should be selected not only based on reprogramming efficiency but also on sample accessibility, clinical applicability, and the intended downstream applications, including disease modeling, regenerative medicine, biobanking, or precision medicine.

#### 3.4.2. Culture Microenvironment

The cellular microenvironment strongly influences reprogramming outcomes. Maintenance and generation of iPSCs require culture conditions that preserve pluripotency while minimizing differentiation, genomic instability, and xeno-contamination. Traditionally, iPSCs have been cultured on mouse embryonic fibroblast (MEF) feeder layers, which provide factors that support self-renewal, but their use introduces animal-derived components and potential xenogeneic contaminants [151,152]. To address these limitations, feeder-free and defined culture systems have been developed, including Matrigel-based and gelatin-based substrates [153,154]. However, Matrigel is derived from mouse sarcoma cells and contains a complex mixture of extracellular matrix proteins and growth factors. Its complex and undefined composition can contribute to batch-to-batch variability, while its animal-derived components may introduce additional variability into the culture system. Such variability may affect the reproducibility and consistency of iPSC maintenance and differentiation outcomes [155]. More recently, recombinant laminin-based platforms such as the laminin-511E8 (iMatrix-511) system combined with defined media (e.g., StemFit™) have enabled efficient feeder-free maintenance of human pluripotent stem cells, although cost and production complexity remain challenges [156].

Beyond culture conditions, additional features of the cellular microenvironment can modulate the efficiency of somatic cell reprogramming [157]. Studies have demonstrated that hypoxic culture conditions enhance iPSC generation, a finding that has been validated in both human and mouse cells [158]. Furthermore, Forristal et al. showed that hypoxia-inducible factors (HIFs) regulate the expression of key pluripotency-associated transcription factors, including OCT4, SOX2, and NANOG, in human embryonic stem cells cultured under low-oxygen conditions [159].

#### 3.4.3. Signaling Pathways Regulating Reprogramming

Cell fate transitions during somatic cell reprogramming are tightly regulated by multiple signaling pathways that coordinate epigenetic remodeling, cellular plasticity, proliferation, and survival. Among these, transforming growth factor-β (TGF-β) signaling represents one of the major barriers to reprogramming by maintaining the mesenchymal phenotype and inhibiting MET, an early and essential step in the acquisition of pluripotency [100,101]. Consequently, inhibition of TGF-β signaling using small molecules such as SB431542 or RepSox promotes MET and significantly enhances reprogramming efficiency [100,160]. In contrast, Wnt/β-catenin signaling exhibits stage-dependent functions during reprogramming. Transient activation of Wnt signaling facilitates the establishment and maintenance of pluripotency by promoting self-renewal and activation of pluripotency-associated genes, whereas inappropriate or prolonged activation during early reprogramming may impair MET and reduce reprogramming efficiency [102,161,162]. Growth factor-mediated pathways also play important regulatory roles. LIF/STAT3 signaling is indispensable for maintaining naïve pluripotency in mouse stem cells, whereas Activin/Nodal and FGF signaling are essential for sustaining human pluripotent stem cells by regulating self-renewal and suppression of differentiation [163,164,165]. In addition, insulin-like growth factor (IGF) signaling enhances cell survival, proliferation, and metabolic adaptation during reprogramming, further improving iPSC generation [151]. Accordingly, optimization of culture media with signaling modulators, including TGF-β inhibitors, Wnt pathway regulators, histone deacetylase inhibitors, and appropriate growth factors, has become an effective strategy to enhance reprogramming efficiency and improve the quality of iPSC lines.

#### 3.4.4. Epigenetic Barriers to Reprogramming

Somatic cell reprogramming is limited by multiple epigenetic barriers that maintain somatic identity and restrict activation of pluripotency networks [157]. A major obstacle is DNA methylation, which suppresses key pluripotency genes [166]; modulation of DNA methyltransferases or activation of TET-mediated demethylation can enhance reprogramming efficiency by promoting gene activation, including OCT4, highlighting the central role of dynamic DNA methylation in pluripotency induction [109,167].

Repressive histone modifications also hinder reprogramming. H3K9 methylation restricts the binding of reprogramming factors to chromatin, whereas inhibition of H3K9 histone methyltransferases or activation of the histone demethylase KDM3A enhances reprogramming efficiency by promoting chromatin accessibility and reactivating pluripotency genes such as *Oct4* [92]. H3K27 methylation further represses pluripotency genes, including Sox2 and Nanog [90,168,169]. In addition, H3K79 methylation maintains somatic transcriptional programs, and its inhibition enhances reprogramming efficiency [106]. Small molecules such as vitamin C and valproic acid enhance reprogramming by modulating epigenetic and cellular pathways. Vitamin C promotes TET-mediated DNA demethylation and histone demethylation, facilitating chromatin remodeling and activation of pluripotency-associated genes. In addition, vitamin C suppresses cellular senescence and oxidative stress, thereby improving cell survival, genomic stability, and overall reprogramming efficiency. In contrast, valproic acid functions as a histone deacetylase inhibitor that increases chromatin accessibility and relieves epigenetic repression, further facilitating induction of pluripotency [118].

#### 3.4.5. p53-Mediated Barriers to Reprogramming

A major limitation of somatic cell reprogramming into iPSCs is its low efficiency, which is largely attributed to activation of the tumor suppressor p53 during the reprogramming process [170]. Because several reprogramming factors are oncogenic, their overexpression triggers p53-dependent responses such as cell cycle arrest, apoptosis, and senescence, while also suppressing pluripotency genes like Nanog, thereby blocking reprogramming [171,172]. As a result, transient inhibition of p53 or its downstream targets (e.g., p21 and Puma), as well as factors that suppress p53 activity such as Oct4 and ZSCAN4, can enhance reprogramming efficiency [172,173,174]. However, because p53 is essential for genomic stability, its suppression during reprogramming is associated with increased genetic abnormalities and potential oncogenic mutations in iPSCs, raising significant safety concerns for clinical applications [170]. For applications requiring clinically relevant iPSC-derived models or cell products, increased reprogramming efficiency alone is therefore insufficient. Fit-for-purpose benchmarks should include genomic stability, absence of potentially oncogenic alterations, maintenance of pluripotency and differentiation capacity, and functional comparison with appropriate primary human cells or tissues. The specific acceptance criteria should ultimately be defined by the intended context of use.

Although p53 is generally regarded as a barrier to transcription factor-mediated reprogramming, recent studies suggest that its role is more complex in chemical-induced pluripotency. Depending on the stage of reprogramming and the signaling pathways involved, p53 may contribute to appropriate epigenetic remodeling and maintenance of genomic integrity, indicating that its function is context-dependent rather than uniformly inhibitory [175].

### 3.5. Characterization and Functional Validation

Rigorous characterization of newly generated iPSC lines is essential to establish their identity, pluripotency, genetic stability, and safety prior to banking and downstream clinical applications. To ensure these quality standards are met, a comprehensive panel of assays is recommended, including pluripotency assessment, differentiation potential testing, genetic analysis, cell identity verification, gene expression profiling, and sterility testing [176,177]. In 2023, the International Society for Stem Cell Research (ISSCR) released updated guidelines aimed at standardizing human stem cell line characterization and promoting greater transparency, rigor, and reproducibility in stem cell research [178].

#### 3.5.1. Morphology

Morphological characterization is a fundamental first step in assessing iPSC quality. Undifferentiated iPSCs typically display small, round cells with a high nucleus-to-cytoplasm ratio, prominent nucleoli, and tightly packed colonies with well-defined borders [179,180]. Because culture conditions can influence morphology, stem cell laboratories are encouraged to provide representative images of both undifferentiated and differentiated cells to facilitate comparison and quality assessment [181]. Phase-contrast microscopy is the primary method for routine assessment [182].

#### 3.5.2. Molecular Characterization

Pluripotency is confirmed by the expression of intracellular markers such as OCT4, NANOG, and SOX2, together with cell surface markers including SSEA3, SSEA4, TRA-1-60, and TRA-1-81, while SSEA-1 is associated with differentiation rather than the undifferentiated state [176,179,181]. For accurate iPSC characterization, it is recommended to assess both intracellular and cell surface pluripotency markers. Acceptable flow cytometry results require ≥70% of cells to express pluripotency markers and ≤10% of cells to express the differentiation-associated marker SSEA-1 [181]. Flow cytometry is the standard quantitative method, complemented by immunocytochemistry and qRT-PCR [181]. Additionally, Alkaline phosphatase (AP) activity is widely used as a marker of pluripotent, undifferentiated stem cells [183]. AP staining provides a rapid, sensitive method for visualizing iPSC-positive colonies [184]. However, AP is not specific to stemness, as it can also be expressed in other cell types and is influenced by differentiation status [183]. These limitations should be considered when interpreting AP staining results.

#### 3.5.3. Genomic Characterization

Because prolonged culture can introduce chromosomal abnormalities, evaluation of genetic stability is considered an essential component of iPSC quality control [185].

G-banding karyotyping remains the standard method for assessing chromosomal integrity and is recommended routinely, typically every 10 passages [177,178,181,186]. It effectively detects large chromosomal abnormalities but has limited resolution for small genomic alterations [186]. Higher-resolution methods, including chromosomal microarray analysis, single nucleotide polymorphism (SNP) arrays, comparative genomic hybridization (CGH), whole-exome sequencing, or whole-genome sequencing, may identify subchromosomal copy number variations and acquired mutations [63,178,187].

#### 3.5.4. Functional Characterization

Functional pluripotency requires demonstration of differentiation into derivatives of all three germ layers (ectoderm, mesoderm, and endoderm) [178].

Embryoid body (EB) formation is the most commonly used spontaneous differentiation assay. Following suspension culture without FGF, PSCs form EBs that differentiate into all three germ layers. Differentiation is confirmed using lineage-specific markers such as TUJ1 (ectoderm), AFP/SOX17 (endoderm), and SMA (mesoderm), typically assessed by immunocytochemistry or RT-PCR [179,181,186].

Directed differentiation uses defined growth factors and culture conditions to generate lineage-specific cell populations. Trilineage differentiation refers to the ability of pluripotent stem cells to differentiate into derivatives of the three embryonic germ layers—ectoderm, mesoderm, and endoderm—and is recommended by the ISSCR for evaluating pluripotency using multiple lineage-specific markers [178,179,181].

Teratoma formation was historically considered the gold standard for pluripotency assessment but is no longer recommended as a routine assay due to ethical concerns, high cost, variability, and reliance on animal models [178,186,188]. Current recommendations favor combining embryoid body formation, directed differentiation, and gene expression analyses as reliable alternatives for functional pluripotency assessment [178,188].

#### 3.5.5. Reproducibility and Functional Relevance of iPSC-Derived In Vitro Models

In addition to assessing pluripotency and genomic stability, rigorous iPSC-based disease modeling requires consideration of cell-line identity, donor and clone variability, and reproducibility across differentiation experiments. Cell-line authentication should be performed to confirm sample identity and minimize the risk of cross-contamination or misidentification [178]. The use of multiple independent donor-derived iPSC lines and, where appropriate, multiple clones from each donor can help distinguish disease-associated phenotypes from donor- or clone-specific effects, provided that their interdependence is appropriately considered in experimental design and statistical analysis [189,190]. For genetically defined disease models, isogenic controls generated by correcting or introducing the disease-associated variant within the same genetic background can further strengthen causal interpretation of observed phenotypes [191].

The clearance of reprogramming vectors should also be evaluated following reprogramming, particularly when integrating or potentially persistent reprogramming methods are used, as residual vector sequences may affect genomic stability [192]. Residual vector sequences can be assessed using vector- or transgene-specific PCR or quantitative PCR, with testing performed after sufficient passages to confirm that vector sequences are no longer detectable [130]. In addition to routine assessment of chromosomal abnormalities, mitochondrial integrity and function should be considered as part of comprehensive iPSC quality control [193].

Validation of differentiated cells should extend beyond the expression of lineage-specific markers. Assessment of cellular maturation is important because iPSC-derived cells may exhibit immature phenotypes relative to their adult tissue counterparts [194]. Therefore, differentiated cells should be benchmarked against primary cells or native tissues using molecular, morphological, and functional measures where appropriate. Importantly, expression of lineage-specific or tissue-specific markers alone is insufficient to establish physiological equivalence to native tissue. Functional validation should incorporate organ- or cell-type-specific endpoints relevant to the model, such as electrophysiological activity, contractility, metabolic function, secretion, or other appropriate physiological responses [194].

## 4. 3D Organoids

A 3D organoid is an in vitro three-dimensional cellular construct derived from stem cells, such as iPSCs or adult tissue-resident stem cells. It possesses the capacity for self-organization and self-renewal, bridging the critical gap between 2D cell cultures and complex living organisms. Conventional 2D monolayer culture constrains cells to a flat, rigid substrate: apical-basal polarity is lost, cell–cell and cell–matrix contacts are largely replaced by contact with tissue-culture plastic, and the oxygen, nutrient, and signaling gradients that pattern native tissue are abolished. Cells maintained under these conditions progressively diverge in morphology and gene expression from their tissue of origin, which limits their predictive value for drug response and toxicity testing. By restoring three-dimensional architecture, cell–cell and cell–matrix interactions, apical-basal polarity, and physiological gradients, 3D organoids address each of these specific shortcomings.

### 4.1. History of Organoid Development

The history of 3D organoid models is a journey from early 20th century observations of cellular self-assembly to the precise, stem-cell-driven “mini-organs” widely used in modern biomedical research (Figure 4). This development integrates advances from developmental biology, stem cell biology, tissue engineering, and biomaterials science, ultimately enabling researchers to recreate organ-like structures in vitro that closely mimic native tissue architecture and function.

The conceptual foundation of organoid biology can be traced back to the early twentieth century. In 1907, Henry Van Peters Wilson demonstrated that dissociated sponge cells could spontaneously reaggregate and regenerate into a functional organism, revealing for the first time that cells possess intrinsic self-organizing properties [195,196]. This pioneering experiment established the principle that cellular organization is not entirely externally imposed but instead can emerge through autonomous cell–cell interactions. Decades later, Steinberg, M.S further expanded this concept through the Differential Adhesion Hypothesis (DAH), proposing that cells sort and spatially arrange themselves according to thermodynamic differences in surface adhesion properties [197,198]. These early discoveries laid the theoretical groundwork for understanding how complex multicellular structures can emerge from relatively simple cellular populations.

Despite these conceptual advances, the field remained technically limited until major breakthroughs in stem cell biology occurred. A transformative milestone was achieved in 1981 with the first successful isolation of embryonic stem cells (ESCs) from mouse embryos by Evans, M.J. and M.H. Kaufman [5], as well as Martin, G.R. independently [6]. These pluripotent cells demonstrated the remarkable ability to self-renew indefinitely while retaining the capacity to differentiate into all embryonic lineages. The later derivation of human embryonic stem cells (hESCs) in 1998 further revolutionized developmental biology and regenerative medicine by providing access to human pluripotent cells for in vitro modeling [7]. Another landmark advance occurred in 2006 when Takahashi and Yamanaka introduced induced pluripotent stem cell (iPSC) technology, demonstrating that differentiated somatic cells could be reprogrammed back into a pluripotent state through the ectopic expression of defined transcription factors [8]. This discovery dramatically expanded the accessibility of patient-specific stem cells and established the foundation for personalized organoid systems.

A major turning point in organoid research occurred in 2009 when Sato et al. from the Clevers laboratory established the first long-term 3D intestinal organoid culture derived from single Lgr5+ adult stem cells isolated from mouse intestinal crypts [9]. By embedding these stem cells in Matrigel and supplying a defined cocktail of niche factors, including R-spondin, Noggin, and epidermal growth factor (EGF), the researchers demonstrated that adult stem cells could self-organize into crypt-villus structures closely resembling native intestinal tissue. This landmark study is widely regarded as the birth of modern organoid technology because it provided a reproducible system capable of long-term expansion while maintaining tissue-specific architecture and functionality.

Following this breakthrough, the 2010s witnessed an explosive expansion of the organoid field. Advances in pluripotent stem cell differentiation protocols, extracellular matrix engineering, and culture systems enabled the successful development of organoid models for numerous human tissues and organs. Cerebral organoids capable of recapitulating aspects of early cortical development were introduced by Lancaster et al., providing unprecedented opportunities for modeling neurodevelopmental disorders [199]. Similarly, liver, pancreas, kidney, lung, retinal, cardiac, gastric, and endometrial organoids were subsequently established from both adult stem cells (ASCs) and pluripotent stem cells (PSCs), greatly broadening the applicability of organoid technologies. During this period, organoids rapidly evolved from relatively simple epithelial structures into increasingly sophisticated multicellular systems incorporating stromal, immune, vascular, and neuronal components.

More recently, the field has advanced toward highly engineered and physiologically relevant systems. Emerging technologies such as organ-on-a-chip platforms [200], 3D bioprinting [201], synthetic extracellular matrices [202], and assembloid engineering [203] now allow precise control over spatial organization, vascularization, and inter-organ communication. In parallel, patient-derived tumor organoids have become powerful tools for precision oncology, enabling individualized drug screening and preservation of tumor heterogeneity. Collectively, these advances have transformed organoids from experimental developmental models into versatile platforms for disease modeling [20], regenerative medicine [204], drug discovery [205], personalized therapeutic applications [206].

Today, organoid technology continues to evolve toward increasingly complex and mature tissue systems that more faithfully recapitulate in vivo physiology. As culture methods, biomaterials, and multi-omics characterization techniques continue to improve, organoids are expected to play an even more central role in translational research and future clinical applications.

### 4.2. Cell Sources and Organoid Generation

Pluripotent stem cells (PSCs) and adult stem cells (ASCs) represent the two most prominent and widely used pillars for establishing 3D organoid systems. PSCs are pluripotent cells capable of differentiating into virtually any cell type of an organism. This remarkable plasticity allows researchers to reconstruct developmental programs in vitro by applying tightly controlled, stepwise differentiation protocols. Typically, this involves the sequential addition of growth factors and small molecules that recapitulate key signaling pathways active during in vivo embryogenesis, including germ layer specification, regional patterning, and tissue morphogenesis. Under these defined conditions, PSCs progressively self-organize into complex three-dimensional structures that resemble the architecture, cellular diversity, and developmental trajectory of native organs.

A classic example is the generation of human brain organoids from hiPSCs. In this system, iPSCs are first aggregated into embryoid bodies (EBs), which serve as early 3D precursors. These aggregates are then directed toward ectodermal lineage specification through exposure to signaling modulators such as Noggin, LDN-193189, and SB431542, which inhibit BMP and TGF-β pathways. Following neural induction, cells differentiate into neuroepithelial structures that further expand and organize into brain-like tissue containing multiple neural cell types, including neurons, astrocytes, and progenitor populations. Over time, these structures undergo maturation processes that recapitulate aspects of early human brain development, including cortical organization and regional identity formation [207].

In contrast, ASC-derived organoids originate from tissue-resident stem cell populations isolated from adult or patient-derived tissues, including both human and animal sources. A well-established example is the generation of mouse intestinal organoids. In this system, intestinal crypts are enzymatically or mechanically isolated and dissociated into small clusters containing resident stem cells. These clusters are then embedded in an extracellular matrix (ECM), typically Matrigel, and cultured in defined media enriched with niche-supporting factors such as R-spondin, Noggin, and Wnt agonists. These signaling components are critical for maintaining Lgr5+ intestinal stem cells in a proliferative and self-renewing state. Under these conditions, the cells spontaneously self-organize into crypt-villus–like structures that recapitulate both the morphology and key functional properties of intestinal epithelium [9].

Beyond iPSC- and ASC-derived systems, additional cell sources and specialized approaches have further expanded the organoid field. Embryonic stem cells (ESCs), for instance, remain the gold standard for developmental biology studies due to their high pluripotency and lack of epigenetic memory associated with reprogramming. In addition, tumor-derived organoids (tumoroids) are widely used in cancer research and precision oncology. These systems preserve the genetic, transcriptomic, and phenotypic characteristics of the original patient tumor, making them powerful tools for drug screening, biomarker discovery, and personalized therapeutic testing while maintaining the heterogeneity of the malignancy. This technology has enabled medical centers to establish functional cancer biobanks using patient-derived organoids, a strategy first successfully demonstrated for prostate and colorectal malignancies [208,209]. Building on these early biobanks, recent advancements have integrated patient-derived tumoroids with emerging engineering platforms, such as microfluidic organ-on-a-chip devices, 3D bioprinting, and complex immune cell co-cultures to accurately model the human tumor microenvironment [210,211].

Beyond cell source diversity, organoid generation strategies can also be broadly classified based on assembly principles. A commonly used approach is the “top-down” strategy, in which stem cells are guided to self-organize into organoids primarily through intrinsic developmental programs and external biochemical cues. This approach leverages the inherent self-organization capacity of stem cells to generate organ-like structures with minimal physical manipulation. In contrast, “bottom-up” strategies rely on the controlled assembly of pre-differentiated or isolated cell populations, such as epithelial, mesenchymal, or endothelial cells. These defined populations are co-cultured to reconstruct tissue architecture in a more engineered and modular manner. Such approaches are particularly important for generating multi-lineage organoids that require precise spatial organization and intercellular communication [212].

To successfully recapitulate complex tissue architectures in vitro, researchers utilize diverse biofabrication strategies designed to precisely manipulate the cellular microenvironment. In terms of methodology, these techniques are broadly categorized into scaffold-based, scaffold-free, and bio-engineered systems. While early organoid platforms relied heavily on naturally derived matrices to guide initial self-assembly, subsequent technological iterations have introduced sophisticated synthetic and engineered environments. Each of these overarching approaches offers distinct compromises between biological complexity and experimental control, directly influencing the structural fidelity, scalability, and ultimate physiological relevance of the resulting model (Table 3).

As detailed in Table 3, selecting an ideal generation methodology requires balancing the specific goals of the downstream application against the inherent limitations of each system. For instance, while scaffold-based systems excel at supporting complex, long-term multi-lineage morphogenesis, their undefined chemical nature poses challenges for high-throughput pharmacological screens where batch-to-batch consistency is paramount. Conversely, scaffold-free techniques sacrifice native extracellular matrix signaling but offer the scalability and morphological uniformity necessary for large-scale drug discovery. More recently, the field has begun transitioning toward hybridized approaches, integrating bio-engineered platforms like 3D bioprinting or microfluidic organ-on-a-chip systems with traditional hydrogels [200,214]. These advanced configurations are intended to circumvent historical limitations by introducing precise spatial control and dynamic fluid flow, thereby allowing researchers to model critical physiological features such as functional vascularization and multi-tissue interfaces [215,216].

### 4.3. Characterization of Organoids

Characterizing 3D organoids is essential to validate that they successfully recapitulate the structural, cellular, and functional properties of the target organs. Because organoids are designed to mimic in vivo tissues, comprehensive multi-level characterization is required to confirm that they not only resemble the correct morphology but also contain the appropriate cell types and exhibit organ-specific physiological functions.

Structural and morphological assessment serves as fundamental approaches for confirming the physical organization, size, and spatial architecture of organoids. This is typically performed using microscopy to monitor growth, symmetry, and overall morphology over time. Confocal microscopy is widely used due to its high spatial resolution, enabling detailed characterization of both whole-mount and sectioned organoids following immunofluorescent staining of lineage-specific or structural markers. More advanced 3D live-imaging modalities, such as light-sheet fluorescence microscopy (LSFM), enable the real-time capture of entire organoid volumes with minimal phototoxicity. Capitalizing on these optical benefits, recent engineering breakthroughs have introduced high-throughput, open-top multiwell light-sheet platforms. These systems facilitate the parallel, long-term imaging of dozens of mature intestinal, hepatic, and tumor organoids simultaneously [217].

At the cellular level, flow cytometry (FACS) is a high-throughput method used to quantify the proportion of different cell populations within dissociated organoids using fluorophore-conjugated antibodies. This platform enables not only the rapid assessment of cellular heterogeneity and differentiation efficiency but also the precise quantification of cell death and therapeutic responses within dense 3D structures [218]. In parallel, single-cell RNA sequencing (scRNA-seq) has emerged as a gold-standard technique for unbiased cellular characterization. It enables comprehensive identification of all cell types within an organoid and provides detailed insight into transcriptomic profiles, lineage relationships, and developmental trajectories. As a result, scRNA-seq is now widely used for validating organoid identity and benchmarking differentiation protocols against in vivo tissue states [219].

While transcriptomic analyses reveal gene expression programs and cellular ‘potential,’ proteomic profiling provides critical, complementary insights into the actual functional state of the model system [220]. Proteomics, particularly mass spectrometry–based approaches, captures real-time changes in protein abundance, post-translational modifications, and secreted factors, thereby offering a more accurate reflection of the active biological processes occurring within the microenvironment [221]. Consequently, this approach is uniquely valuable for evaluating whether developing organoids have achieved a mature, physiologically relevant state that faithfully recapitulates native tissue function.

Beyond molecular profiling, functional characterization is critical to truly ‘perform’ like their in vivo counterparts. Functional assays assess physiological outputs that are specific to each organ system. For example, electrophysiological recordings, including multi-electrode arrays (MEA), are used to measure neuronal firing patterns in brain organoids and contractile activity in cardiac organoids [222]. In epithelial systems such as intestinal organoids, fluorescent dextran permeability assays are commonly used to evaluate barrier integrity and epithelial permeability [223]. In hepatic organoids, quantification of albumin secretion and urea production serves as a key indicator of metabolic and synthetic liver function [212]. Similarly, secretion-based assays (e.g., ELISA) are used across multiple organoid types to quantify cytokines, hormones, or other functional outputs.

Together, these complementary approaches provide a multi-dimensional framework for organoid characterization, spanning morphology, cellular composition, molecular state, and functional performance. The information each assay provides, together with its principal limitations, is summarized in Table 4. Because these levels address different questions, the assays are complementary rather than interchangeable, and a characterization strategy is best assembled by selecting across levels rather than by choosing the single most informative technique. Assembling such a strategy is not, however, the same as validating the model. Characterization becomes validation only when its outputs are judged against criteria defined in advance. Marker expression alone is not evidence of physiological equivalence: a cell expressing a lineage marker may still lack the transporter activity, secretory output, or electrophysiological behavior of its in vivo counterpart. A defensible strategy therefore states which of the organ-specific endpoints described above must be met, and what quantitative thresholds constitute acceptance, rather than reporting only that an assay was performed. It also requires benchmarking against native tissue rather than against other in vitro models, since maturation is the most common point of divergence: organoids frequently arrest at a fetal-like transcriptional profile, and stating maturity explicitly allows a reader to judge fitness for a given context of use. Finally, batch-level criteria are needed alongside per-organoid ones. Size, shape and cellular composition vary between organoids within a batch and between batches, and this variability is compounded by the undefined, lot-variable composition of naturally derived matrices such as Matrigel [202]. Defining batch acceptance criteria-the proportion of organoids meeting morphological and functional thresholds, and the matrix lot used is a prerequisite for reproducibility across laboratories.

### 4.4. Types of Organoids and Their Applications

Recent advances in stem cell biology, tissue engineering, and organoid culture technology have enabled the development of a wide variety of organoid systems that reproduce selected aspects of the structural organization, cellular heterogeneity, and physiological functions of native human tissues and organs. Through the combination of pluripotent stem cell differentiation, adult stem cell expansion, and optimized extracellular matrix–based culture systems, researchers are now able to generate highly complex three-dimensional in vitro models that mimic key aspects of organ development, tissue homeostasis, and disease progression. As a result, organoids have rapidly emerged as one of the most transformative platforms in modern biomedical research, bridging the gap between conventional two-dimensional cell culture and in vivo animal models.

Currently, organoids have been successfully established for numerous organ systems, including the intestine, brain, liver, lung, kidney, pancreas, stomach, heart, retina, skin, and endometrium (Table 5). Each organoid type is designed to reproduce tissue-specific architecture and function while maintaining relevant cellular diversity. Importantly, these systems provide a physiologically relevant microenvironment that allows cells to self-organize and interact in ways that closely resemble in vivo biology. Compared with traditional monolayer cultures, organoids better preserve tissue polarity, cell–cell communication, and developmental signaling pathways. The extent of this advantage is tissue- and protocol-dependent, however, and has been established more thoroughly for some organ systems than for others; improved architectural fidelity should not be assumed to confer improved predictive accuracy without direct benchmarking against the relevant in vivo endpoint.

Among the earliest and most extensively studied systems are intestinal organoids, which were first established from Lgr5+ intestinal stem cells [9]. These organoids recapitulate the crypt-villus organization of the intestinal epithelium and contain multiple differentiated cell lineages, including enterocytes, goblet cells, Paneth cells, and enteroendocrine cells. Intestinal organoids are now widely used to investigate epithelial homeostasis, stem cell maintenance, nutrient absorption, and host–microbiome interactions. In addition, they have become valuable platforms for modeling inflammatory bowel disease (IBD) [224], intestinal infections [225], and colorectal cancer [209], as well as for evaluating drug absorption and metabolism [226].

Cerebral or brain organoids represent another major breakthrough in organoid research. Derived primarily from pluripotent stem cells, these systems mimic key aspects of early human brain development, including neural progenitor expansion, cortical layer formation, and regional specification. Brain organoids have provided unprecedented opportunities for studying neurodevelopmental disorders such as microcephaly [199], autism spectrum disorders [227], epilepsy [227], as well as neurodegenerative diseases [228]. More advanced neural assembloid systems now allow the investigation of inter-regional neuronal connectivity and neural circuit formation, significantly expanding the relevance of organoids for neuroscience research [229,230].

In parallel, liver, kidney, and lung organoids have become increasingly important platforms for disease modeling, regenerative medicine, and pharmaceutical applications [231,232,233,234]. Liver organoids are widely used to study metabolic disorders, viral hepatitis, fibrosis, and hepatotoxicity due to their ability to reproduce key hepatic functions such as albumin secretion and drug metabolism [235]. Kidney organoids containing nephron-like structures have enabled the study of kidney development, renal fibrosis and its therapeutic targets, and a widening range of renal disease applications [234,236,237]. Similarly, lung organoids mimicking airway and alveolar structures have become highly valuable for investigating respiratory infections, including influenza and SARS-CoV-2 [232], as well as chronic lung diseases such as fibrosis and cystic fibrosis [238].

Additional organoid systems have further broadened the scope of the field. Pancreatic organoids are used to study diabetes, pancreatic development, and pancreatic ductal adenocarcinoma (PDAC) [239], while gastric organoids provide models for studying Helicobacter pylori infection and gastric cancer [240]. Cardiac organoids are increasingly utilized for investigating heart development, cardiac disease modelling, and drug evaluation, with recent systems achieving vascularized, chamber-like structures containing cardiomyocytes, endothelial cells, and fibroblasts [241,242]. Retinal organoids recapitulate layered retinal organization and are used for vision research, inherited retinal disease modeling, and gene therapy testing [243,244]. Endometrial organoids have become powerful tools for studying hormone responsiveness, embryo implantation, infertility, and endometriosis [245,246]. More recently, skin organoids capable of forming stratified epidermal structures and appendage-like tissues have emerged as promising models for wound healing, regenerative medicine, and dermatological disease studies [204,247].

Among the most clinically relevant applications are tumor-derived organoids (tumoroids), which are generated directly from patient tumor samples. These organoids retain many of the genetic, transcriptomic, and phenotypic characteristics of the original tumors, including intratumoral heterogeneity and patient-specific drug responses. Consequently, tumor organoids have emerged as powerful tools for cancer biology research, precision oncology, biomarker discovery, immunotherapy testing, and personalized medicine [206,248]. Because they preserve clinically relevant tumor behavior (organoid drug responses are benchmarked against the same patient’s actual treatment response) more effectively than conventional cancer cell lines, tumoroids are increasingly being explored as predictive platforms for individualized therapeutic decision-making.

Collectively, the rapid expansion of organoid technology has substantially broadened the range of human-relevant in vitro models available across multiple organ systems. As culture methodologies, biomaterials, vascularization strategies, and multi-cellular engineering techniques continue to improve, organoids are expected to play an increasingly important role in developmental biology, disease modeling, drug discovery, regenerative medicine, and future translational and clinical applications.

**Table 5:** Different Types of Organoids and Their Applications.

Organoids Type	Application	References
Intestinal organoids	Studying homeostasis and microbiome interactions; disease modeling; drug screening	[9,20,205]
Brain organoids	Neurodevelopment studies; neurodegenerative disease modeling; neurotoxicity testing	[203,207,249,250]
Liver organoids	Liver disease modeling; drug-induced liver injury; virus infection; regenerative medicine; transplantation research	[212,231,233,251,252]
Lung organoids	Respiratory infections; cystic fibrosis; lung cancer; drug screening	[232,238,253,254]
Kidney organoids	Nephrotoxicity screening; kidney development; chronic kidney disease model	[234,236,237]
Pancreatic organoids	Diabetes modeling; Pancreatic development; pancreatic cancer; drug screening	[239,255]
Heart organoids	Cardiotoxicity testing; heart development; heart disease modeling	[241,242]
Endometrial organoids	Infertility studies; endometriosis studies; reproductive toxicity testing	[245,246]
Skin organoids	Wound healing; skin regeneration; infection and skin disease modeling; bioprinting	[204,247]
Tumor-derived organoids	Precision oncology; drug response prediction; tumor heterogeneity study	[256,257,258]

### 4.5. Challenges in Organoid Systems and Strategies to Overcome Them

Despite their revolutionary impact on biomedical research, organoid systems still face a fundamental hurdle: they are not complete, intact systems like their in vivo counterparts. While they successfully recapitulate the 3D cellular architecture and specific functional lineages of native tissues, current organoids lack the holistic systemic integration required to fully mimic a living organism. The most prominent shortcomings stem from a lack of critical physiological components, such as:

Vascularization: Without a functional, perfusable blood vessel network, organoids rely purely on passive diffusion for nutrient supply and waste removal, which inherently limits their growth and leads to a necrotic core as they increase in size. To address the absence of a vascular network, researchers are employing two primary strategies: either differentiating hiPSCs into endothelial cells to co-culture them with other cell types differentiated from hiPSCs to form the organoids [259,260], or driving the co-differentiation of endogenous vascular lineages directly within the developing organoid itself [261].

Immune Microenvironment: Most standard organoid models lack resident and circulating immune cells (such as microglia in brain organoids or macrophages in gut organoids), leaving them unable to simulate complex inflammatory or host-defense responses. Researchers have embedded immune cells such as macrophages, natural killer (NK) cells, or engineered CAR-T cells into the extracellular matrix alongside the organoids. This approach is highly effective for observing targeted interactions, such as studying how macrophages drive tissue inflammation or measuring the direct cytotoxicity (cell-killing ability) of T cells against a specific tissue target [262].

Inter-organ Crosstalk: As isolated structures, organoids cannot reproduce the intricate multi-organ communication, such as the gut-liver-brain axis that dictates drug metabolism, systemic endocrine regulation, and holistic disease progression. To overcome the lack of inter-organ crosstalk, researchers are developing assembloids, in which the integrated structures are formed by merging distinct region-specific organoids (such as different brain regions) [203]; assembloids spanning different organ systems remain an emerging goal. Also, to bridge these systemic gaps, researchers are increasingly turning to advanced bioengineering platforms, most notably Organ-on-a-Chip technologies [263] and microfluidic multi-organ systems. These platforms place micro-scale tissues inside engineered devices lined with fluid channels, allowing scientists to actively pump nutrient media and simulate the mechanical forces of blood flow [216]. A further consideration is that these engineered platforms specify the physical microenvironment quantitatively rather than leaving it to passive diffusion, which is what makes them complementary to organoids rather than simply more complex. In the Exoliver liver-on-a-chip platform, for example, perfusion is ramped from a wall shear stress of 0.1 to 1.15 dyn/cm^2^ (1.5 mL/min) over the first 2 h of culture, matching an organ that physiologically receives approximately 25% of resting cardiac output and has a normal tissue stiffness of 400–600 Pa, rising to 1.2–1.6 kPa in fibrosis [215]. Oxygen tension can likewise be imposed as a defined variable rather than left at ambient: combined bone and bone marrow organoids are maintained at 5% O_2_ for the first 20 days to reflect the hypoxic marrow niche, then transferred to 20% O_2_ for a further two weeks to mature lymphoid and myeloid output [264]. Reporting such parameters explicitly, rather than describing culture conditions qualitatively, is a prerequisite for reproducibility across laboratories and for the benchmark datasets that regulatory acceptance of these models will require.

## 5. Conclusions

In vitro cellular models have evolved from primary cells and established cell lines to iPSC systems, organoids, and increasingly complex bioengineered platforms. Each model provides distinct advantages and limitations, and greater complexity does not necessarily translate into greater experimental or translational value. Rather, model selection should be guided by the biological question, intended context of use, and an appropriate balance among biological relevance, reproducibility, scalability, and experimental control. Importantly, rigorous characterization, standardization, and fit-for-purpose validation will be essential for translating these technologies into reliable in vitro models. Together, these complementary approaches provide an evolving framework for improving disease modeling, drug development, safety assessment, and ultimately the translation of in vitro findings to human biology.

## 6. Future Directions

Cell culture has evolved from primary cells, which preserve many native tissue features but have limited lifespan and variability, to established cell lines, which provide scalable, reproducible, and experimentally tractable systems for mechanistic studies. These models have supported basic discoveries in cell biology, genetics, infection, cancer, toxicology, and pharmacology. More recently, advanced in vitro systems, including iPSC-derived organoids, organ-on-chip platforms, and other New Approach Methodologies (NAMs), have expanded cell culture toward more human-relevant biology. Together, these platforms form a continuum from discovery models to translational tools for disease modeling, drug development, safety testing, and precision medicine (Figure 5).

This direction is strongly reinforced by recent U.S. policy, the FDA Modernization Act 2.0, by allowing the use of non-animal approaches for drug development, followed by a roadmap and draft guidance for NAMs, including organoids, organ-on-chip systems, in silico models (AI-based tools), and other validated human-relevant platforms [265,266,267]. The NIH Complement-ARIE program focuses on developing, standardizing, and validating NAMs to better model human health and disease. FDA and NIH are actively working together to advance regulatory documents and promote acceptance of these methods [268,269,270]. FDA’s 2026 draft guidance highlights a validation framework based on context of use, relevance, robustness, reproducibility, and standardization [269]. This direction is especially important but more complex for brain organoids, which may improve modeling of human neurodevelopment, neurotoxicity, infection, and degenerative diseases. However, regulatory acceptance will require clear quality control standards, defined maturation stages, benchmark datasets, reproducible protocols, and functional readouts. This policy shift should not be interpreted as automatic validation or regulatory acceptance of organoids or other NAM platforms. Regulatory applicability remains dependent on the intended context of use and evidence demonstrating biological relevance, reproducibility, robustness, and appropriate performance for the specific application.

On the other hand, AI and machine learning (ML) are increasingly being integrated across the in vitro model-development workflow, from culture optimization to organoid characterization and predictive modeling. AI-enabled image analysis can automate organoid segmentation, tracking, morphological classification, and longitudinal assessment using label-free bright-field or three-dimensional imaging, thereby reducing operator-dependent variability and enabling higher-throughput phenotypic analysis. For example, deep-learning platforms such as OrganoID and OrBITS can quantify organoid size, morphology, growth dynamics, and treatment responses from time-resolved images, supporting objective assessment of culture quality and drug response [271]. When integrated with automated culture systems, continuous imaging and ML-based classification may further enable non-destructive quality control, early identification of abnormal differentiation or growth, and selection of organoids meeting predefined quality attributes [272]. Beyond imaging, ML can analyze multidimensional culture parameters to optimize complex media formulations and culture conditions more efficiently than conventional empirical approaches [273]. AI approaches can also integrate imaging with transcriptomic, proteomic, metabolomic, and functional datasets to identify phenotypic signatures and develop predictive models of organoid development or therapeutic response. Ultimately, coupling AI with automated culture, real-time imaging, and multi-omics could facilitate adaptive, data-driven culture systems while improving scalability, reproducibility, and standardization. However, robust training datasets, external validation, model interpretability, and cross-laboratory generalizability will be essential before AI-derived outputs can serve as reliable quality-control or translational endpoints.

A more speculative but important frontier is organoid intelligence [274,275,276,277], which combines brain organoids, electrophysiology, microfluidics, and machine interfaces to study learning, computation, neurotoxicity, and disease-relevant neural function. While still early and ethically sensitive, organoid intelligence highlights the broader future of cell culture: not merely growing cells but building dynamic human-relevant biological systems that can model development, disease, drug response, and even complex information processing. Ethical oversight is expanding, particularly for advanced brain organoids and organoid intelligence systems that interface with electrodes, receive inputs, or generate neural activity outputs. Thus, the future direction is not simply replacement of animal models, but disciplined integration of brain organoids into validated NAM pipelines for human-relevant neuroscience, drug safety, and disease modeling.

Organ-on-chip and microphysiological systems provide dynamic control of biomechanical and physicochemical parameters that are difficult to reproduce in conventional static cultures. Perfusion can be adjusted to generate tissue-specific fluid shear stress, while channel dimensions, flow rates, and perfusion conditions regulate nutrient transport and biochemical gradients. Importantly, appropriate shear stress varies substantially among tissues and vascular compartments and therefore should be selected based on the physiology being modeled. Oxygen tension can also be controlled to reproduce physiological or pathological microenvironments, including hypoxic conditions, while integrated sensors can enable real-time monitoring of oxygen and other environmental parameters. Thus, flow rate, shear stress, oxygen tension, channel geometry, and cell density should be quantitatively defined and optimized according to the tissue model and intended context of use, improving physiological relevance, reproducibility, and standardization of organ-on-chip systems [278,279].

Last but not least, standardization and validation are distinct but interdependent processes rather than strictly sequential steps. Initial proof-of-concept validation may precede extensive standardization, while standardization establishes consistent procedures, quality-control criteria, and analytical endpoints that enable more rigorous and reproducible validation. For advanced in vitro models and NAMs, these processes should therefore be viewed as iterative, ultimately supporting validation within a defined context of use.

NAMs are likely to become an increasingly important platform for future transplantation-based regenerative therapies, although their clinical use will probably emerge first in selected tissues rather than as whole-organ replacements. Because organoids can be generated from adult stem cells or patient-derived iPSCs, they offer the possibility of producing expandable, tissue-specific grafts for repairing damaged epithelium, restoring endocrine function, or replacing localized tissue defects. Intestinal organoid transplantation, for example, is already being explored as a regenerative strategy for inflammatory bowel disease, with experimental studies showing tissue repair and functional recovery, and early human trials beginning to emerge. Similarly, pancreatic islet organoids are being investigated as potential transplantable constructs for diabetes, especially when combined with vascularization, encapsulation, immune protection, and alternative implantation-site strategies. However, broad therapeutic application will require solutions to major translational barriers, including long-term engraftment, vascular and neural integration, immune compatibility, genetic and epigenetic stability, tumorigenicity, reproducible manufacturing, xeno-free matrices, and regulatory quality control (QC). Therefore, the most realistic prediction is that organoids will first serve as localized regenerative grafts or supportive cell-based implants, while more complex organ replacement will require further advances in bioengineering, biomaterials, perfusion systems, and immune modulation.

## Figures and Tables

**Figure 1:**
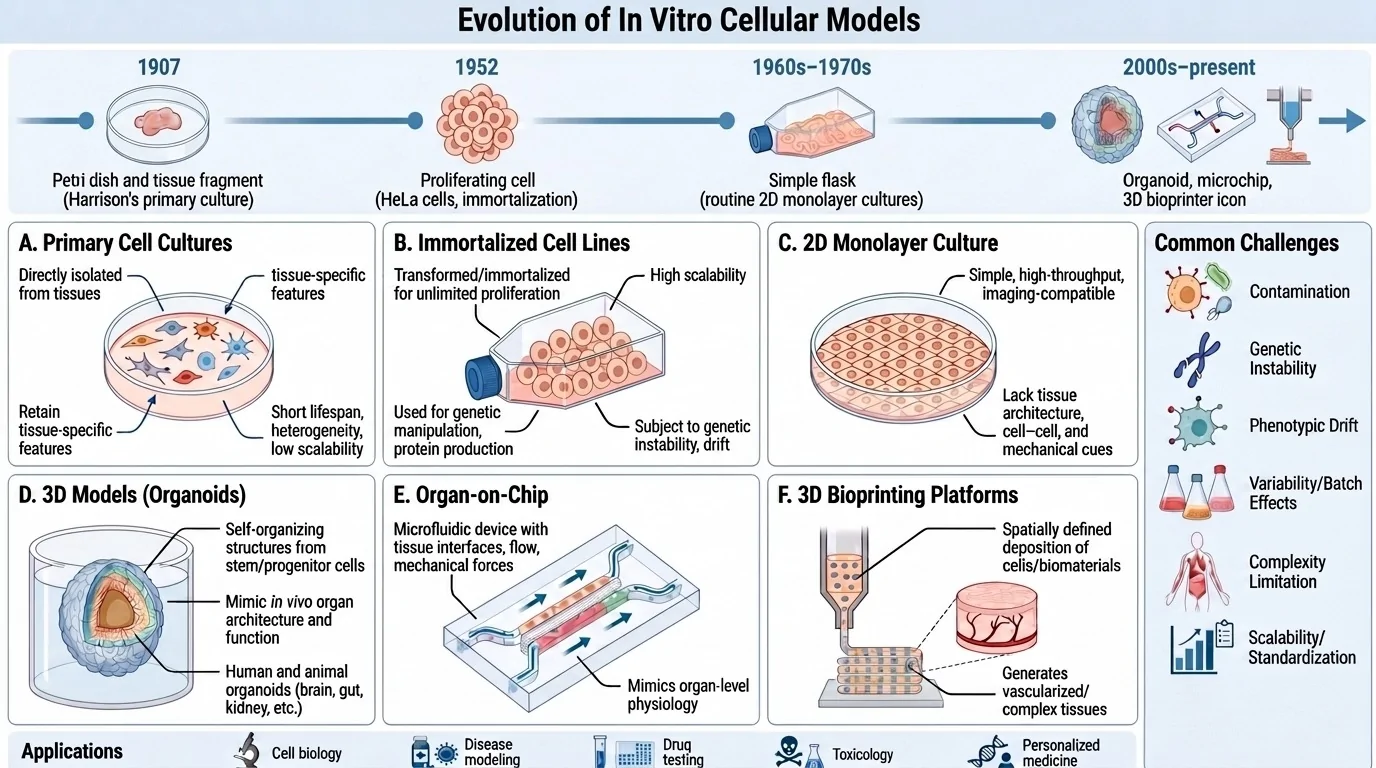
Evolution of in vitro cellular models. The figure highlights key milestones in the development of cell culture and modern in vitro models and their potential applications, as well as common limitations. Created with Figurelabs.

**Figure 2:**
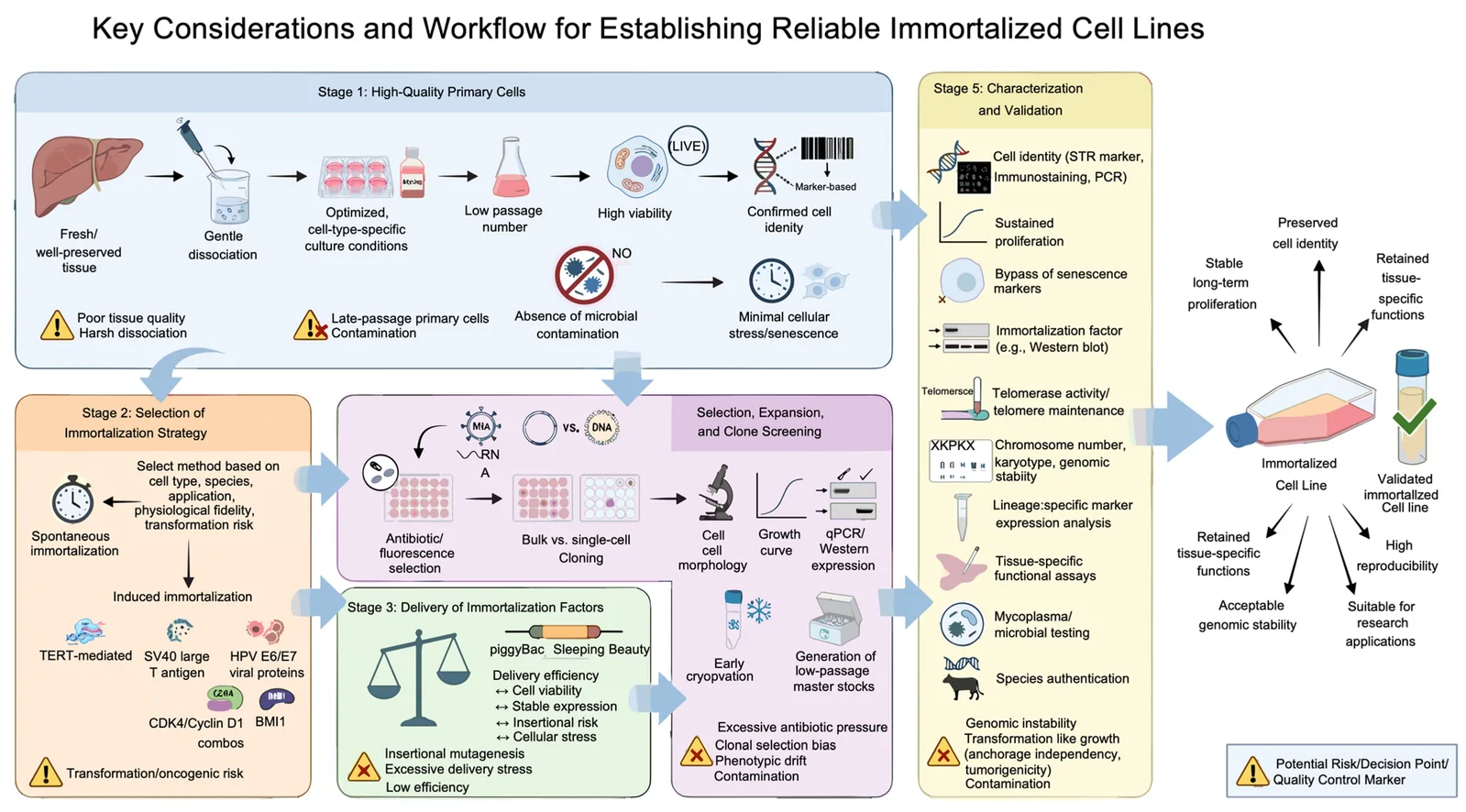
Key Considerations and Workflow for Establishing Reliable Immortalized Cell Lines. Successful immortalization requires a stepwise approach beginning with healthy, early-passage primary cells and optimization of culture conditions prior to immortalization. The choice of immortalization strategy should be guided by the biological characteristics of the target cells and the intended downstream applications. Comprehensive validation, including assessment of cellular identity, molecular fidelity, and cell type–specific functions relative to parental primary cells, is essential. Routine cryobanking and transparent reporting of experimental procedures further ensure the reliability, reproducibility, and long-term utility of immortalized cell lines. Created with Figurelabs.

**Figure 3:**
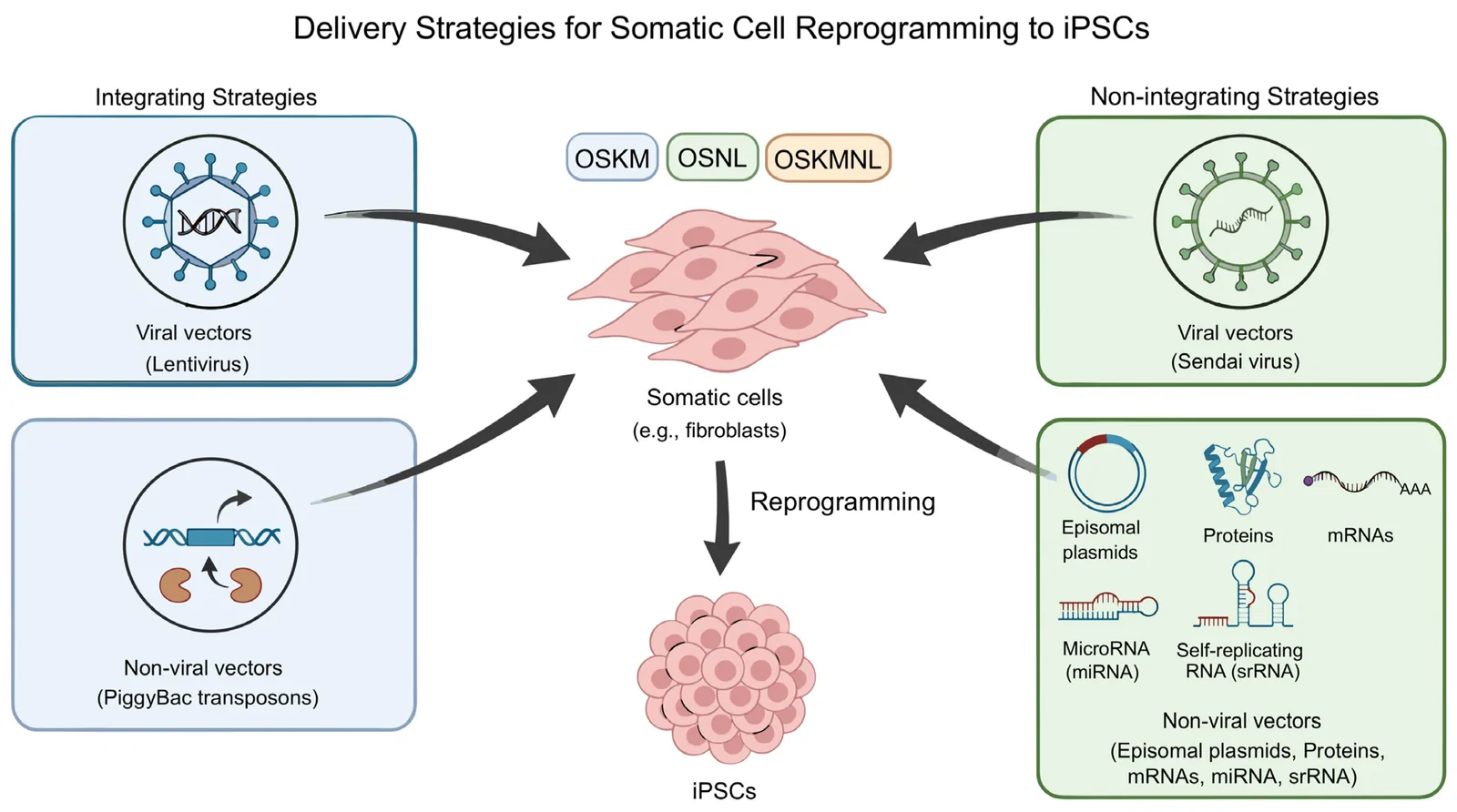
Reprogramming strategies used to generate induced pluripotent stem cells (iPSCs). Overview of integrating and non-integrating approaches used for iPSC reprogramming using different combinations of reprogramming factors, including OSKM (OCT4, SOX2, KLF4, and c-MYC), OSNL (OCT4, SOX2, NANOG, and LIN28), and OSKMNL (OCT4, SOX2, KLF4, c-MYC, NANOG, and LIN28). These approaches promote the reprogramming of somatic cells toward a pluripotent state. Created with FigureLabs.

**Figure 4:**
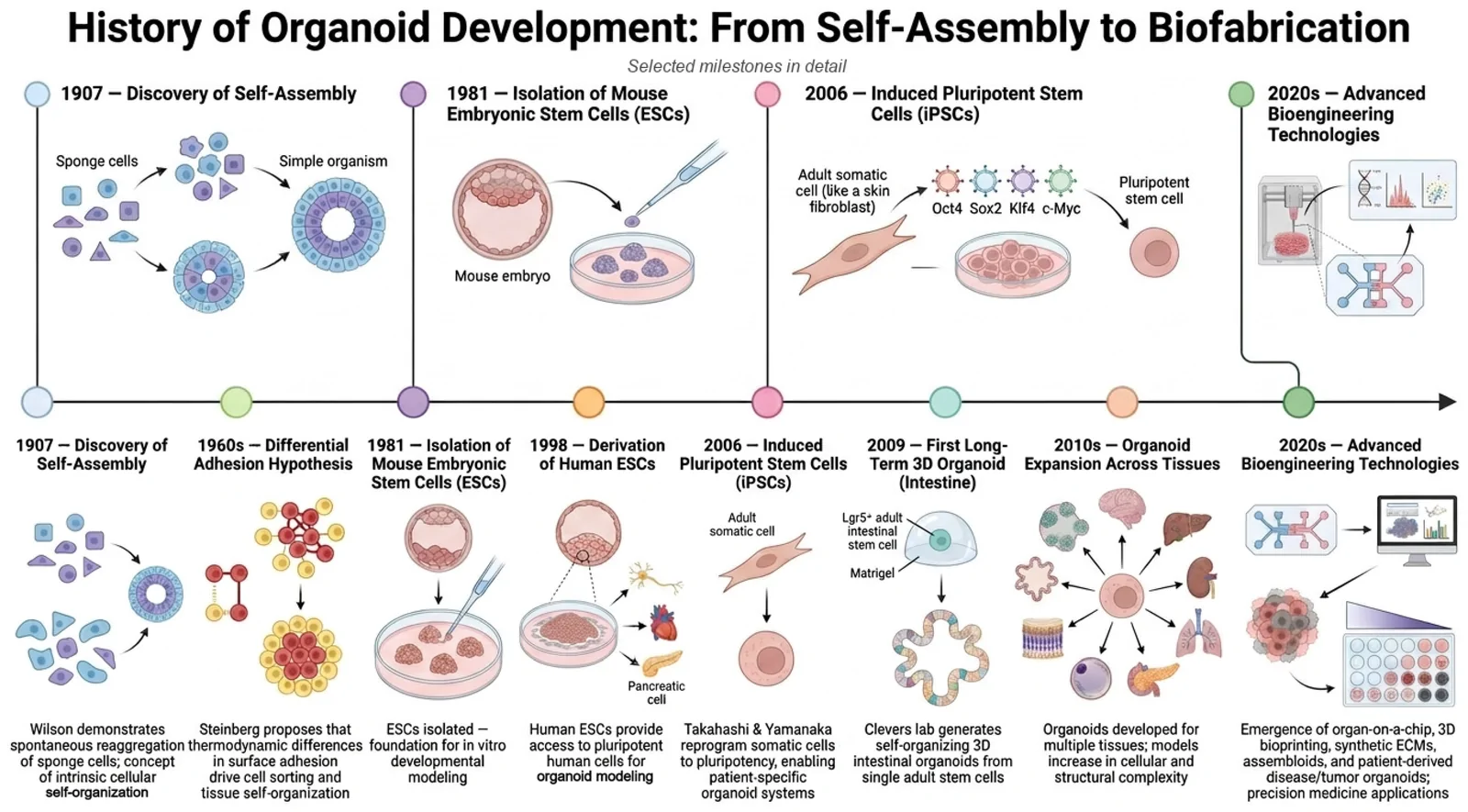
Historical timeline of 3D organoid development. The timeline outlines the progressive evolution of cellular models, beginning with early observations of cell self-assembly and leading up to advanced biofabrication technologies. Created with Figurelabs.

**Figure 5:**
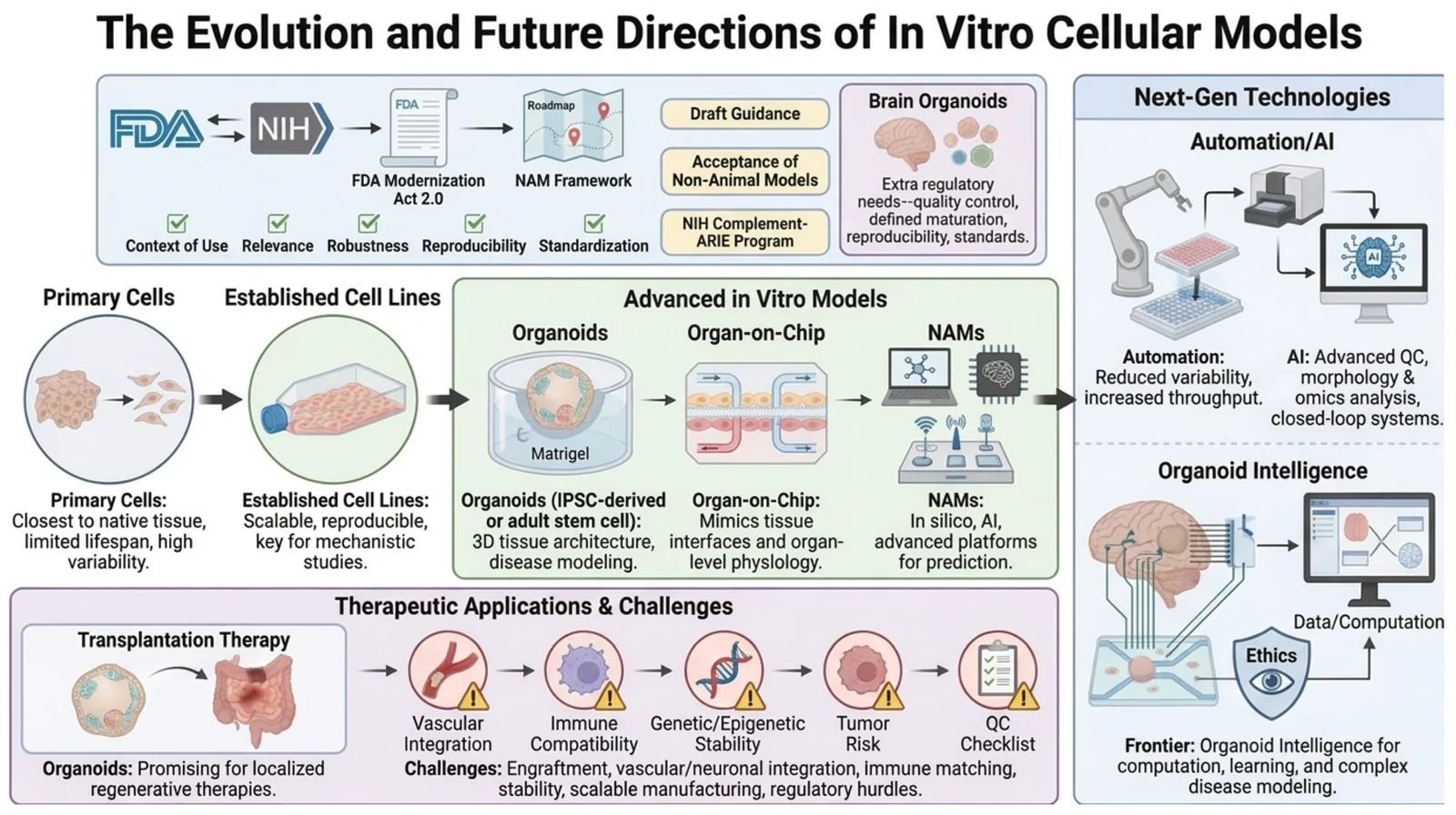
Evolution and future directions of in vitro cellular models. The figure shows the progression from primary cells and established cell lines to organoids, organs-on-chip, and other NAMs. Future advances will rely on regulatory standardization, automation, AI, and improved quality control. Major translational challenges include tissue integration, immune compatibility, genetic stability, scalability, and tumor risk. Created with Figurelabs.

**Table 1:** Comparative Summary of Major In Vitro Cellular Model Systems.

Model Type	Key Strengths	Key Limitations	Ideal Applications
Primary cells	Directly derived from native tissues; can retain important tissue-, donor-, and species-specific molecular and functional characteristics; no immortalization-associated manipulation	Finite proliferative capacity; donor/animal variability; potential cellular heterogeneity; limited scalability; phenotype and function may change during prolonged culture; culture requirements are often cell-type specific	Studies of native cellular physiology and function; donor- or species-specific biology; disease modeling; host–pathogen interactions; toxicology and pharmacology; validation of findings obtained using established cell lines
Immortalized cell lines	Extended proliferative capacity; readily expandable and scalable; amenable to repeated experiments and genetic manipulation; can provide greater experimental consistency under standardized conditions	Immortalization and clonal selection may alter genotype, phenotype, signaling, or function; genetic/epigenetic drift may occur during prolonged culture; fidelity to parental cells depends on cell type and immortalization strategy	Mechanistic studies; genetic manipulation; assay development; high-throughput screening; long-term or repeated experiments; applications requiring large and relatively consistent cell numbers
iPSCs	Long-term self-renewal and pluripotency; can generate diverse differentiated cell types; enables donor-/patient-specific models; compatible with genome editing and isogenic model generation; provides access to otherwise difficult-to-obtain human cell types	Reprogramming can be inefficient and heterogeneous; donor and clone variability; residual epigenetic memory; potential genetic and mitochondrial alterations; differentiation efficiency and maturity can vary; extensive QC and validation required	Developmental studies; patient-specific disease modeling; genetic disease modeling; drug discovery and toxicity testing; precision medicine; regenerative medicine; generation of specialized human cell types
Organoids	3D self-organization; can reproduce important aspects of tissue architecture, cellular diversity, polarity, and organ-specific functions; patient-derived models can retain disease- or tumor-specific features; supports multicellular and spatial interactions not captured by conventional 2D culture	Batch and protocol variability; dependence on culture conditions and ECM/matrix; variable cellular composition and maturation; limited vascularization, immune components, innervation, and systemic interactions in many models; relatively high cost and technical complexity; scalability and standardization remain challenging	Developmental and tissue-biology studies; complex disease modeling; patient-derived tumor modeling and precision oncology; drug efficacy and toxicity testing; host–pathogen studies; regenerative medicine; studies requiring tissue architecture or multicellular interactions

**Table 2:** Comparison of Common Somatic Cell Sources for iPSC Reprogramming.

Cell Source	Accessibility	Reprogramming Efficiency	Advantages	Limitations
Fibroblasts	Skin biopsy	Moderate	Well established	Invasive; slower
PBMCs	Blood draw	Moderate–High	Minimally invasive	Heterogeneous
Keratinocytes	Skin/hair	High	Fast reprogramming	Requires epithelial culture
Urine-derived cells	Non-invasive	Moderate–High	Easy collection	Variable yield
Cord blood cells	Banking	High	Young cells	Limited availability
Dental pulp stem cells	Tooth	High	Highly proliferative	Limited source
MSCs	Bone marrow/adipose	High	Multipotent	Donor variability

**Table 3:** Summary of Methodologies for Generating 3D Organoids.

Approach	Techniques	Advantages	Challenges
Scaffold-based	Hydrogel embedding (e.g., Matrigel, synthetic ECM) [9] embryoid body aggregation followed by Matrigel embedding [207]	Mimics native extracellular matrix; supports complex 3D morphogenesis and long-term culture	Batch-to-batch variability; limited spatial and mechanical control; undefined composition in natural ECMs
Scaffold-free	Hanging drop; forced aggregation in microwell arrays [213]	High uniformity of organoid size; scalable and cost-effective; defined culture conditions	Lack of ECM support; reduced physiological relevance; limited structural complexity
Bio-engineered	3D bioprinting [201]	High spatial and temporal control; enables vascularization and multi-tissue integration; advanced physiological modeling	Technically demanding; higher cost; requires specialized equipment and optimization

Organ-on-a-chip and microfluidic systems are culture and perfusion platforms rather than methods of organoid generation, and are therefore not listed here; they are discussed in Section 4.5.

**Table 4:** Assays for the multi-level characterization of 3D organoids.

Level	Assay	Information Obtained	Principal Limitations
Morphological	Light and fluorescence microscopy; confocal and light-sheet microscopy	Three-dimensional architecture, organoid size and symmetry, marker localization, and growth followed over time in the same sample	Deep imaging requires clearing or sectioning; throughput is limited by acquisition and image-analysis time
Cellular	Flow cytometry (FACS)	Quantitative proportions of defined cell populations, together with cell death and therapeutic response within dissociated organoids	Requires dissociation to single cells, so spatial information is lost; restricted to markers selected in advance
Cellular	Single-cell RNA sequencing	Unbiased identification of the cell types present, their lineage relationships and developmental trajectories, without prior marker selection	Higher cost and analytical burden than flow cytometry applied to the same samples; dissociation bias; yields neither spatial nor protein-level information
Molecular	RT-qPCR	Abundance of selected transcripts; rapid confirmation that lineage-specific markers are expressed	Limited to pre-selected targets and provides no spatial context; transcript abundance alone does not establish that the corresponding protein is produced
Proteomic	Mass spectrometry	Abundance of proteins, their post-translational modifications and the secreted factors actually present. Because transcript abundance correlates only partially with protein output, this measures the functional state that transcriptomic assays can predict but not confirm	Higher sample input and cost than transcript-level assays addressing the same pathway; data interpretation is complex
Functional	Fluorescent dextran permeability	Epithelial barrier integrity and luminal transport	Applicable only to organoids with an enclosed lumen; results depend on organoid size and shape, and are difficult to quantify in closed spheres
Functional	Multi-electrode array (MEA)	Real-time electrical activity, including neuronal firing patterns and cardiac excitability	Applicable only to electrically active tissues; requires specialized equipment and is highly sensitive to the culture environment
Functional	Secretory assays (e.g., ELISA)	Quantification of specific secreted outputs such as albumin, urea, cytokines or hormones	Reports a population average with no attribution to individual cells; each assay measures one analyte

Assays are grouped by the level of organoid biology they interrogate. Techniques within a group address comparable questions and may to some extent be substituted for one another; techniques in different groups are complementary rather than interchangeable. Cost and duration are therefore stated only relative to alternatives within the same group.

## Data Availability

No new data were created or analyzed in this review. Data sharing is not applicable to this article.
